# A framework for structuring Thai medical documents using systematized nomenclature of medicine clinical terms and large language models

**DOI:** 10.1371/journal.pdig.0001791

**Published:** 2026-10-30

**Authors:** Nat Tangchitnob, Wanchana Ponthongmak, Boonchai Kijsanayotin, Oraluck Pattanaprateep, Sithakom Phusanti, Pongsakorn Atiksawedparit, Kamonporn Suwanthaweemeesuk, Gareth J. McKay, Takehiko Oami, Ammarin Thakkinstian

**Affiliations:** 1 Department of Clinical Epidemiology and Biostatistics, Faculty of Medicine Ramathibodi Hospital, Mahidol University, Bangkok, Thailand; 2 Thai Health Information Standards Development Center, Health Systems Research Institute, Ministry of Public Health, Bangkok, Thailand; 3 Faculty of Medicine Ramathibodi Hospital, Chakri Naruebodindra Medical Institute, Mahidol University, Bangkok, Thailand; 4 Centre for Public Health, Queen’s University Belfast, Belfast, United Kingdom; 5 Department of Emergency and Critical Care Medicine, Chiba University Graduate School of Medicine, Chiba, Japan; Indiana University Indianapolis, UNITED STATES OF AMERICA

## Abstract

Systematized Nomenclature of Medicine – Clinical Terms (SNOMED CT) enables semantically interoperable health data, but implementation in Thailand is hindered by language and workflow challenges. We evaluated whether large language models (LLMs) can automate SNOMED CT concept extraction from Thai clinical documents. The objectives of this study are to develop a proof-of-concept framework for automated clinical named entity recognition and SNOMED CT concept linking from Thai medical documents, and to compare the performance and resource requirements of closed-source and open-source LLMs on a human-annotated dataset. The dataset was manually annotated from 220 Thai medical documents (6,390 entities) from three outpatient departments. Our retrieval-augmented framework was evaluated using four LLMs (Gemini 2.5 Pro, GPT-4.1, Qwen 3, and MedGemma) as experimental based models which were comparatively analyzed for entity recognition using precision, recall, F1 score and concept linking using character-level metric, Intersection over Union (IoU), as well as resource consumptions. For entity recognition, Gemini achieved the highest performance (loose F1 83.31%). For SNOMED CT concept extraction ranked most similar (Top-1) by encoder models for retrieval and re-ranking, IoU scores were 39.42% (Gemini), 34.44% (GPT), 29.86% (MedGemma), and 28.62% (Qwen). Average processing time for end-to-end extraction was 5–6 minutes per document for Gemini and GPT, with cost estimates in United states dollars (USD) of USD 0.035 and USD 0.019 per document, respectively; local open-source inference required up to 10 minutes per document. This study established a proof-of-concept framework for automated clinical entity recognition and SNOMED CT concept linking from Thai medical documents, demonstrating practical LLM performance with trade-offs between accuracy, cost, and efficiency across closed-source and open-source models.

## Author summary

We developed and tested a way to turn Thai clinical notes into a standard structured health data. This work addresses a problem in healthcare data landscape where the most useful clinical information is recorded in the messy doctor notes making it difficult to use, analyze, and share for research and decision-making. This challenge is particularly more pronounced in non-English speaking settings like Thailand where fewer tools and resources are available. With the advancement of language model technologies, we used large language models to automatically identify medical concepts in Thai medical documents and link them to standardized international medical terminology. The results show promise, but performance depends on how clearly information is written and how well local terms match standard terms. We found that the language technologies can capture clinical concepts in low-resourced language settings. In long term, this may support better secondary use of health data which is the foundational requirement for data-driven decision-making, patient-centered healthcare delivery, and value-based health system.

## Introduction

Semantic interoperability represents the ability for heterogeneous healthcare systems to exchange and consistently interpret data and represents a major challenge for modern digital healthcare provision. Achieving semantic interoperability typically requires standardized clinical terminologies. Systemized nomenclature of medicine–clinical terms (SNOMED CT) is one of the most comprehensive international terminologies for representing clinical concepts into a machine-readable form. Although SNOMED CT offers a robust framework for data exchange and broad concept coverage for standardized representation of clinical information, including the Thai healthcare system [1,2], it lacks the mechanism for capturing the full context of clinical data from diverse medical documents, especially unstructured free text [3,4].

In routine practice, medical documents are typically produced as free-text narratives entered directly by clinical practitioners rather than encoded using standardized terminologies. Such notes often contain abbreviated expressions, incomplete sentences, and idiosyncratic phrasing, that depend on shared contextual knowledge among clinical practitioners [5–7]. This challenge is amplified in bilingual clinical contexts, such as in Thailand, which is further exacerbated by hybrid technical terms, local abbreviations, and acronyms [2,8]. These disparities across different specialized users, domains, and languages pose a major barrier to the wider adoption and utility of SNOMED CT [9].

The advancement of modern language technologies has enabled automatic extraction and annotation of clinically relevant information from free text health records. Named entity recognition (NER) is a foundational task in clinical natural language processing that identify mentions of clinical entities in free-text documents and categorize them to the predefined clinically meaningful domains (such as medication, procedure, laboratory test, and clinical finding). These identified entities are subsequently mapped to semantically equivalent concepts in a standardized clinical terminology, known as clinical entity linking; together, the combined workflow is commonly referred to as clinical NER and entity linking (NER + L) [10,11]. Several approaches have been proposed which primarily target English language biomedical text, ranging from rule-based and traditional natural language processing (NLP), (e.g., MetaMap [12] developed and maintained by the U.S. National Library of Medicine), to neural approaches such as bidirectional encoding representation from transformer (BERT) based concept annotation (e.g., MedCAT [13]), which can be adapted to other languages depending on the availability and suitability of the underlying BERT model. However, only general domain BERT tools are currently available that are limited by an absence of Thai medical data for fine-tuning purposes [8,14].

Recently, generative large language models (LLMs) have demonstrated utility across a broad range of NLP tasks, including multilingual processing, with substantial demonstration of medical knowledge without the necessity for task-specific fine-tuning in many settings [15]. Thus, LLMs have been proposed for a wide range of possible healthcare applications, including NER and NER + L [16]. These models served as a catalyst to investigate the process automation of both NER and NER + L for low-resource languages in the clinical domain where the use of conventional NLP tools remain somewhat limited [17,18].

LLMs can achieve competitive performance even without task-specific fine-tuning for clinical domain-level NER. In English language clinical corpora, closed-source models such as GPT-4 have been reported to reach an F1 score of 90.67% [19], with further improvements achieved through structured prompting and verification strategies (e.g., 91.76%) [20]. However, performance is generally much poorer for open-source models and varies across languages and datasets. For example, one study evaluating open-source LLMs reported that Llama-3-8B achieved markedly different F1 scores across different languages with 65.20%, 47.80%, and 22.60% for French, English and Spanish, respectively [17].

With the addition of entity linking (NER + L), the system must not only detect spans and clinical domains but also annotate each entity to a unique concept identifier within a target terminology (e.g., SNOMED CT). This additional requirement typically increases task difficulty with concomitant performance reduction. For instance, an LLM-based approach evaluated on the SNOMED International endorsed dataset reported an intersection over union (IoU) score of 37.77% [11]. Furthermore, LLMs may generate inaccurate or even falsified codes, highlighting the risk of hallucinated structured outputs [21]. By way of mitigation, many pipelines constrain generation by first retrieving candidate concepts from the target terminology and then selecting from those candidates, rather than freely generating identifiers [22,23]. This technique is closely aligned with a retrieval-augmented generation (RAG) approach that seek to ground model outputs and reduce hallucinations [24].

Robust evaluation of non-English clinical NER and NER + L systems requires custom human-annotated corpora developed for each target language [10,17]. However, Thai clinical NER and NER + L have not been systematically evaluated to the best of our knowledge, largely due to the lack of local entity-level annotated datasets for Thai clinical documents. Creating such human-annotated datasets is resource-intensive, requiring significant clinical knowledge, familiarity with clinical terminologies, and strong proficiency in local language and documentation conventions [10,18].

To address this gap, we developed an expert-led, human-annotated dataset and used it to rigorously evaluate both closed and open-source LLMs on clinical NER and NER + L tasks. This study also established a proof-of-concept framework for these tasks within a Thai healthcare context to provide a practical framework for the application and assessment of LLM-based NER and NER + L in a non-native English-speaking clinical setting.

## Results

A total of 6,390 entities were annotated from the 220 documents sampled. Of these, 690 entities were used to develop the framework including prompt engineering, while 5,700 entities served as the final evaluation of the test dataset. Dataset characteristics are described (Table 1), the median (IQR) of entities/document was 23 (IQR: 14.00-39.50). Clinical domains were dominated by findings (n = 3,250 [50.86%]), followed by laboratory (n = 1,055 [16.51%]), procedure (n = 1,005 [15.73%]), and medication (n = 524 [8.20%]). The mapping between these clinical domains and their corresponding semantic tags is shown in Fig 1 and, in further detail is provided in S1-S2Tables.

**Table 1:** Dataset characteristics.

Characteristics	Development	Testing	Total
Documents, n	20	200	220
Entities, n	690	5,700	6,390
Entities/document, median (IQR)	31.5 (18.50-43.75)	22.5 (14.00-38.25)	23 (14.00-39.50)
Word/document, median (IQR)	202 (102.50-289.25)	140 (75.00-249.25)	147.5 (76.75–254.25)
Ratio of Thai language, median (IQR)	0.39 (0.23-0.58)	0.47 (0.26-0.65)	0.46 (0.25-0.65)
Top diagnoses in ICD chapters			
Chapter I, n (%)	7 (35.00)	31 (15.50)	35 (17.27)
Chapter R, n (%)	6 (30.00)	26 (13.00)	32 (15.55)
Chapter E, n (%)	2 (10.00)	28 (14.00)	30 (13.64)
Department			
Family medicine, n (%)	2 (10.00)	34 (17.00)	36 (16.36)
Emergency, n (%)	6 (30.00)	36 (18.00)	42 (19.09)
Internal medicine, n (%)	12 (60.00)	130 (65.00)	142 (64.55)

IQR: Interquartile range, ICD: International Classification of Diseases.

**Fig 1:**
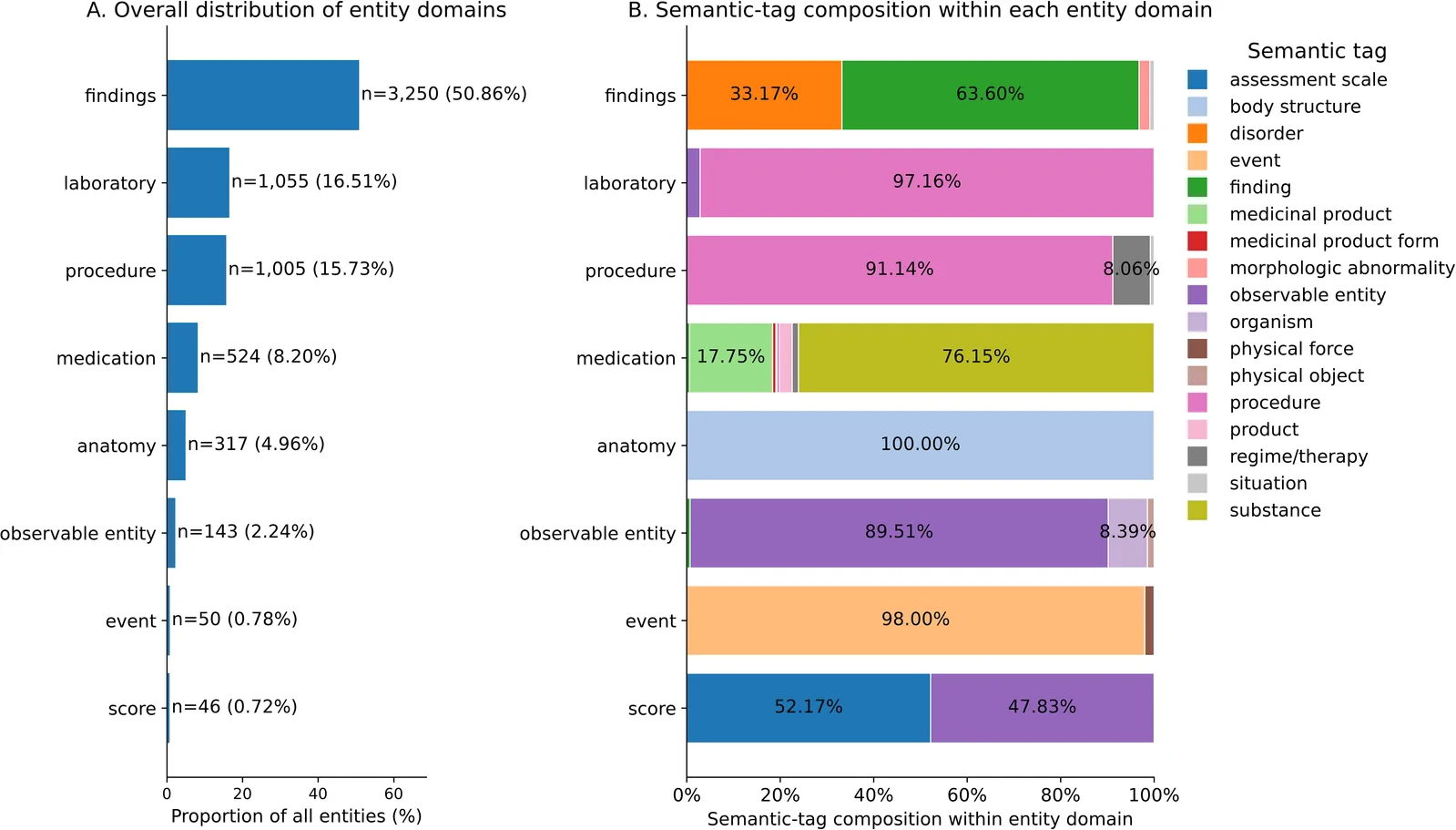
A) Overall distribution of entity domains, B) Semantic tag composition within each entity domain.

### NER performance

The comparative performance of the NER task is summarized in Table 2. Under strict matching criterion, Gemini 2.5 Pro demonstrated the highest recall (70.44%), followed by GPT-4.1 (63.55%), MedGemma (47.22%), and Qwen (44.82%). Gemini also had the highest precision (76.36%) and F1 score (73.28%), followed by MedGemma (71.65%, 56.93%) whereas Qwen demonstrated the lowest performance (51.18% and 47.79%).

**Table 2:** Four LLMs’ performative results and resource consumption requirements.

	Gemini 2.5 pro	GPT 4.1	Qwen 3	MedGemma
NER				
Entity, n (%)	5,259 (92.02)	5,165 (90.38)	4,992 (87.35)	3,750 (65.62)
Strict criteria				
Precision, % (95% CI)	76.36 (75.22 - 77.51)	70.15 (68.90 – 71.39)	51.18 (49.80 – 52.57)	71.65 (70.21 – 73.09)
Recall, % (95% CI)	70.44 (69.26 – 71.63)	63.55 (62.30 – 64.80)	44.82 (43.53 – 46.11)	47.22 (45.92 – 48.52)
F1 score, % (95% CI)	73.28 (72.46 – 74.11)	66.69 (65.80 – 67.57)	47.79 (46.84 – 48.74)	56.93 (55.93 – 57.92)
Loose criteria				
Precision, % (95% CI)	86.92 (86.01 – 87.83)	85.87 (84.92 – 86.82)	80.53 (79.43 – 81.63)	86.77 (85.69 – 87.85)
Recall, % (95% CI)	79.98 (78.95 – 81.02)	77.60 (76.52 – 78.68)	70.34 (69.16 – 71.53)	57.04 (55.76 – 58.33)
F1 score, % (95% CI)	83.31 (82.61 – 84.00)	81.53 (80.80 – 82.25)	75.09 (74.27 – 75.91)	68.83 (67.90 – 69.77)
NER + L (using Top-1)				
Mean IoU score, %	39.42	34.44	28.62	29.86
Strict criteria				
Precision, % (95% CI)	46.53 (45.18 – 47.88)	40.63 (39.29 – 41.97)	28.73 (27.47 – 29.98)	42.83 (41.24 – 44.41)
Recall, % (95% CI)	42.92 (41.64 – 44.21)	36.80 (35.55 – 38.05)	25.15 (24.03 – 26.28)	28.17 (27.00 – 29.34)
F1 score, % (95% CI)	44.65 (43.72 – 45.58)	38.62 (37.70 – 39.53)	26.82 (25.98 – 27.66)	33.99 (33.03 – 34.94)
Hierarchical-aware criteria				
Precision, % (95% CI)	56.97 (55.63 – 58.31)	55.95 (54.59 – 57.30)	44.73 (43.35 – 46.11)	55.97 (54.38 – 57.56)
Recall, % (95% CI)	52.42 (51.13 – 53.72)	50.55 (49.25 – 51.85)	39.07 (37.81 – 40.34)	36.73 (35.48 – 37.98)
F1 score, % (95% CI)	54.60 (53.67 – 55.53)	53.11 (52.17 – 54.05)	41.71 (40.78 – 42.65)	44.35 (43.35 – 45.35)
Average time per document, minute	5.56	5.12	10.62	12.04
Average cost per document, USD	0.035	0.019	–	–
NER + L (using LLM selection)				
Mean IoU score, %	–	37.41	33.54	33.09
Strict criteria				
Precision, % (95% CI)	–	41.72 (40.38 – 43.07)	30.52 (29.23 – 31.80)	42.99 (41.40 – 44.57)
Recall, % (95% CI)	–	37.80 (36.54 – 39.06)	26.35 (25.20 – 27.49)	28.33 (27.16 – 29.50)
F1 score, % (95% CI)	–	39.67 (38.75 – 40.58)	28.28 (27.42 – 29.13)	34.15 (33.20 – 35.11)
Hierarchical-aware criteria				
Precision, % (95% CI)	–	55.41 (54.06 – 56.77)	45.81 (44.42 – 47.21)	56.14 (54.55 – 57.72)
Recall, % (95% CI)	–	50.08 (48.78 – 51.38)	39.46 (38.19 – 40.72)	36.90 (35.65 – 38.15)
F1 score, % (95% CI)	–	52.61 (51.67 – 53.55)	42.40 (41.46 – 43.34)	44.53 (43.53 – 45.53)
Average time per document, minute	–	6.14	14.83	20.47
Average cost per document, USD	–	0.063	–	–

CI: confidence interval, LLM: large language model, NER: named entity recognition, NER + L: named entity recognition and linking, USD: united stated dollar.

Under the loose matching criteria, Gemini 2.5 Pro achieved the highest recall (79.98%), followed by GPT-4.1 (77.60%), Qwen (70.34%) and lowest in MedGemma (57.04%).

Model sensitivity was reflected in the total output volume: Gemini 2.5 Pro identified 5,259 entities (the closest to the number of ground truth entities [5,700 entities]), followed by GPT-4.1 (5,165), Qwen (4,992), and MedGemma (3,750).

### NER + L performance

NER + L performance was evaluated using IoU, strict matching criteria, and hierarchical-aware matching (Table 2). Gemini 2.5 Pro achieved the best overall performance with an IoU of 39.42% and strict recall/precision/F1 of 42.92%/46.53%/44.65%, improving to 52.42%/56.97%/54.60% under hierarchical-aware evaluation.

GPT-4.1 followed (IoU 34.44%) with strict recall/precision/F1 of 36.80%/40.63%/38.62% and hierarchical-aware recall/precision/F1 of 50.55%/55.95%/53.11%. MedGemma showed imbalanced performance, with relatively high precision (42.83%) but low recall (28.17%), and F1 of 33.99% under strict criteria; but 55.97%, 36.73%, and 44.35% under hierarchical-aware respectively. Qwen demonstrated the worst performance across all metrics, with an IoU of 28.62%, strict recall/precision/F1 of 25.15%/28.73%/26.82% and a hierarchical-aware recall/precision/F1 of 39.07%/44.34%/41.71%.

With respect to the LLM-based concept selection, GPT-4.1 marginally improved (IoU 37.41%); strict recall/precision/F1 37.80%/41.72%/39.67%; hierarchical-aware 50.08%/55.41%/52.61%. MedGemma achieved an IoU of 33.09%, with strict recall/precision/F1 of 28.33%/42.99%/34.15% and hierarchical-aware results of 36.90%/56.14%/44.53%, respectively. Qwen improved with an IoU of 33.54%, yielding a strict recall/precision/F1 of 26.35%/30.52%/28.28% and a hierarchical-aware of 39.46%/45.81%/42.40%, respectively. Gemini 2.5 Pro was not evaluated under this setting due to Application Programming Interface (API) rate limits.

### Resource consumption

Resource consumption metrics are presented in Table 2, encompassing total processing time, average time per document, total cost, and average cost per document. Under a Top-1 concept selection, Gemini 2.5 Pro required 5.56 minutes per document at an estimated average cost of United state dollar (USD) 0.035 per document, while GPT-4.1 required 5.12 minutes per document at an average cost pf USD 0.019 per document. Qwen and MedGemma were slower, requiring 10.62 and 12.04 minutes per document, respectively. Under the LLM concept selection, GPT-4.1 required 6.14 minutes per document (USD 0.063 per document), whereas Qwen and MedGemma required 14.83 and 20.47 minutes per document, respectively.

### LLM error analysis

Discrepancy analysis between LLM outputs and the established ground-truth for the test dataset considered the following errors.

#### Textual and linguistic factors.

The idiomatic nature of Thai clinical documentation is characterized by shorthand and high degrees of implicit context. First, the clinical shorthand implicit semantics (e.g., “นอนราบได้” (able to lie down)) is a term implying absence of orthopnea, but for which the models occasionally misclassified as “Events” or “Positions” rather than to the “Clinical Findings” domain. Second, for the multi-component entity, models sometimes struggled to correctly decompose the SNOMED CT concepts. For example, for the phrase “Alcoholic cirrhosis child C,” the LLMs often failed to separately capture the “Child-Pugh score class C (finding),” typically omitting the severity component entirely. Finally, complex syntactic decomposition proved difficult when models attempted to isolate discrete clinical entities from dense narrative strings, such as the failure to segment educational and counseling instructions into individual procedural entities.

#### LLM interpretation errors.

Several error patterns were identified in relation to over-generation and entity omission:

- Entity structural variation: Models, particularly Gemini, often collapsed multiple discrete findings into a single entity (e.g., menstruation attributes such as interval, duration, and amount) leading to false negative (FN) instances under strict evaluation.
- Procedure vs. laboratory confusion: Concepts such as “Hb typing” were inconsistently classified between procedure and lab domains despite correct SNOMED CT linking, reflecting linguistic ambiguity and interpretation errors.
- Contextual leakage: LLMs occasionally embedded modifiers within the entity span (e.g., “Amlodipine 5mg” instead of the substance “Amlodipine”), leading to span and mapping mismatches.

#### SNOMED CT–related challenges.

Many discrepancies were rooted in the ontological structure of SNOMED CT rather than model failure:

- Redundancy: Overlapping concepts [e.g., “NIHSS” (National Institutes of Health Stroke Scale) belonged to both the SNOMED CT’s “Assessment Scale” and “Observable Entity” hierarchies]. Because only one concept could be annotated as the gold-standard concept, clinically valid mappings due to the alternative concept were classified as incorrect under the strict evaluation criterion, resulting in inconsistent scoring.
- Granularity: Models often selected clinically related but more or less specific “neighbor” concepts. For example, “นอนไม่หลับ” (cannot sleep) was mapped to “insomnia” instead of the more specific symptom concept “cannot sleep.” These clinically related mappings were classified as errors under strict matching but were better captured by the hierarchical-aware evaluation, which showed a marked improvement in performance.
- Coverage: The absence of specific SNOMED CT codes for local herbal medicines and certain clinical scores forced models to map some entities to broader, or less specific useful parent concepts. For example, the cultural concept “เห็ดหลินจือ” (Lingzi), a medicinal herb, was mapped to “Mushroom (substance)”; while “croup score” was mapped to the unspecific “Assessment scale (assessment scale)” concept. These examples illustrate how limitations in terminology coverage can affect model performance even when the predicted concepts are clinically or semantically related to the target concepts.

## Discussion

### Principal findings

This study developed and evaluated an end-to-end proof-of-concept framework for Thai clinical NER + L to SNOMED CT concepts using a retrieval-augmented framework with bi-encoder retrieval and cross-encoder re-ranking. Closed-source models, particularly Gemini 2.5 Pro, demonstrated superior and more consistent performance, showing higher robustness to Thai clinical narratives. Model performance was somewhat diminished for concept linking highlighting pipeline sensitivity to upstream factors, especially entity span and normalization terms. Models with high strict NER precision exhibited downstream degeneration, underscoring the importance of accurate boundary detection and limitations with clinical shorthand and ambiguity. In terms of concept linking, the consistent improvements of LLM-selection compared to Top-1 approach were observed across three evaluated models despite Gemini exclusion due to API rate limits. This supports the conclusion that additional LLM-selection step can improve the performance at the cost of increased resource expenditure.

Open-source models exhibited clear trade-offs. Qwen achieved higher loose-match F1 but lower strict precision due to false positive (FP) and boundary errors. In contrast, MedGemma produced higher precision but substantially lower recall, indicating a more conservative extraction behavior and missed entities. Importantly, concept linking further amplified the upstream challenges including boundary inconsistencies and normalization ambiguity.

Resource profiling indicated a practical trade-off between performance and operational cost. LLM-based selection improved candidate ranking but increased computational and orchestration burden. API-based models faced cost and rate-limit constraints for exhaustive selection strategies in real-world deployments, whereas local open-source inference avoids per-request fees but required greater infrastructure and runtime resources. However, with the open-source models’ performance which may have been affected by quantization choices, the processing time comparisons with closed-source models are hardware-dependent and may not generalize to other computational environments.

Error analysis revealed three primary challenges: linguistic ambiguity related to Thai clinical documentation, LLM interpretation errors (e.g., omissions, hallucinations, and contextual leakage); and ontological gaps in SNOMED CT. These discrepancies often arose not only from model limitations, but from inherent difficulties in the mapping of complex Thai clinical language to structured hierarchical terminology. For example, the phrase “นอนราบได้” literally means “can lie flat” and, in Thai clinical context, implies absence of orthopnea. However, models often misinterpreted this expression as event or body position. Similarly, nested clinical expressions introduced ambiguity in entity detection. LLMs sometimes labeled these expressions as single entity, whereas, according to the guideline, these should be separated into multiple entities. These cases suggest that some errors under strict evaluation may represent clinically plausible alternative interpretations rather than model failures. However, incorporating entirety of the guideline instructions into the prompt would substantially increase input token length which increase operational cost and may not necessarily improve model performance.

Beyond annotation and prompt issues, the gap in existing SNOMED CT concepts also contributed to model failures. For example, some entities (e.g., “croup score”) were not presented in the SNOMED CT terminology, resulting in the lack of a correct answer among the candidate concepts to the LLM selection. In such cases, the LLM should be able to recognize that none of available candidates is appropriate match. This highlights the need for a “no match” option to prevent the LLM from being forced to select an incorrect candidate when the correct concept is not available. Mismatch between international standards and local practices (e.g., herbal medicine and clinical scores) further highlights a critical need for localized interface terminologies and the potential benefits of national editions of SNOMED CT. The ultimate achievement of semantic interoperability in the context of Thailand, a low-resource environment, requires a multi-faceted approach: refining human annotation guidelines, enhancing retrieval re-ranking strategies, minimizing model operational costs, and expanding local clinical terminology coverage to better reflect real-world settings.

### Comparison with prior work

The NER performance observed in this study (specifically Gemini 2.5 Pro loose F1 score of 83.31%) strongly aligns with international reports while extending these trends to the nuances of Thai clinical context. In English language medical datasets, high F1 scores of 90.67% was achieved using GPT-4 with self-verification and few-shot examples [19], reaching 91.76% with further integration of retrieval and chain-of-thought [20]. More recently, GPT-4o achieved an F1 score of 83.06% across five clinical domains, which improved to 87.10% when fine-tuned with a small samples of annotated documents [25]. Another study reported F1 scores by GPT-3.5 (79.43%) and GPT-4 (86.07%) on an English language clinical note dataset represented by a limited number of clinical domains: medical problem, treatment, and test [26].

However, direct comparisons across studies must be interpreted with caution. Unlike English corpora, Thai clinical text introduces additional challenges, such as the absence of explicit word boundaries and frequent code-switching, which complicate the extraction process. Despite these linguistic barriers, our open-source models (Qwen 3 and MedGemma) outperformed previous study using open-source multilingual LLMs on European clinical datasets (i.e., English, French, and Spanish languages) with F1 scores of 25.20-47.80%, 43.30-65.20%, and 22.60-43.70%, respectively [17]. Our findings regarding MedGemma’s recall deficit mirror observations from other non-English settings, suggesting that the scarcity of language-specific pre-training remains a bottleneck for model sensitivity in localized medical contexts [17].

A substantial reduction in model performance was observed when the full NER + L pipeline was evaluated, with Gemini 2.5 Pro achieving an IoU score of 39.42%. This result compares favorably to the 37.77% IoU reported on the SNOMED International endorsed dataset using a fine-tuned Mistral model with a vector database [11]. The slight performance gain seen in our study was most likely due to the more advanced modally approach used. Furthermore, our open-source models reached IoU scores between 33.09% and 33.54%, despite the added linguistic difficulty of Thai and without fine-tuning due to the limited availability of Thai–SNOMED CT training corpus.

In the context of limited resource language settings, a study comparing NER using an unsupervised n-gram method reported an F1 score of 42.34% on a German language electronic healthcare record dataset. The F1 score dropped to 19.70% when concept linking was added [23]. A Greek language study adopted the majority vote architecture from LLMs (Gemma and Gemini) to extract entities which were then machine-translated into English and linked to less granular International Classification of Diseases, 10^th^ revision (ICD-10) codes through dense vector retrieval using the BM25 model, followed by re-ranking using MedCPT. The F1 scores for NER and NER + L tasks were 57.61% and 53.39%, respectively [22]. Our results show the empirical efficacy of the open-source LLMs and BERTs without fine-tuning for NER and concept linking, informing the current capability of readily usable models in Thai language, a low-resourced language setting.

The error patterns observed in this study closely mirror findings from those previously reported in prior clinical NLP studies [11,27,28]. As reported with many corpora, structurally mismatched annotations were also observed with documents annotated in parallel by humans, which were in need of further intervention to resolve discrepancies [3,11]. The presence of linguistic ambiguities also align with observations in other language settings [27]; however, idiomatic symptom descriptions and culturally specific expressions, which complicate entity boundaries and concept identification, were particularly pronounced in the Thai language. This is due to the language’s lack of sentence and word boundaries [29], exaggerated by a grammatically incorrect writing style commonly used in medical records [6]. Finally, limitations related to SNOMED CT have also been documented, including redundancy, semantic proximity among parent-child concepts, and incomplete coverage of local concepts [30]. In conclusion, the performance decline observed between NER to NER + L was expected given the constraints imposed by model ability and limitations in the accuracy of the extracted entity and the availability of the target ontology concepts.

### Framework implications

Our pipeline aligns with the global trends toward retrieval-augmented frameworks at an architectural level with similar findings to the SNOMED CT Entity Linking Challenge [11] and combined bi-encoder and cross-encoder concept linking [23,31]. This study suggests that standalone language models are insufficient for classification within highly granular medical ontologies. Instead, the integration of retrieval-augmented and other augmentative approaches are critical for improved performance. The substantial gains observed from re-ranking, especially when employing multilingual general-domain models like bge-large, are consistent with the evidence from a German language study [27] and dictionary-infused RAG approach [24], where vector-based retrieval combined with LLM reasoning or re-ranking offers marked improvements over retrieval alone.

Furthermore, the pre-defined hierarchical structure of SNOMED CT can be exploited to enhance the precision of linking beyond simple lexical similarity. This is evidenced by the performance increase under hierarchical-aware evaluation (10–17% from strict evaluation for all models). This is particularly relevant because the recent large-scale evaluations of SNOMED CT entity linking reported the decreased performance for rare and ambiguous concepts, promoting approaches that incorporate ontology structure and concept definition to support disambiguation and improve robustness [11]. One promising direction is graph-assisted refinement during re-ranking, which has shown improved mapping performance. However, prior work also suggests that aggressive graph expansion (e.g., deeper sampling of hierarchical neighbors) or the use of weakly annotated training data may yield limited gains, indicating that graph assistance should be applied conservatively and subjected to empirical evaluation [31].

Beyond entity spans, modifiers such as laterality, severity, temporality, and negation were also identified in our pipeline. Normalizing this information into structured attributes can further improve concept selection and reduce ambiguity among closely related concepts. This extension aligns with SNOMED CT post-coordination feature, a mechanism to represent improved clinical meaning through the combination of multiple concepts. Recent work has demonstrated the feasibility of an automated post-coordination method, suggesting a pragmatic pathway to extend our framework. Future improvement of our framework could therefore integrate graph-assisted candidate refinement, attribute normalization to support post-coordination, and terminology-service validation to ensure syntactic and semantic correctness for downstream interoperability.

### Strengths

One key strength of this study is the high ecological validity of its data source. By utilizing unstructured free-text narratives captured during actual clinical encounters, the framework was tested against the true “noise” of real-world daily practice rather than synthetic corpora. This offers a more accurate reflection of results that are directly grounded in the practical complexities of real-world medical documentation. Moreover, the terminology development process integrated clinical expertise with advanced NLP techniques within a resource-constrained setting, providing practical insights applicable to similar environments.

Although limited by annotation methodology, the resulting dataset represents the first Thai language medical corpus specifically designed for NER and integrated NER + L tasks to the best of our knowledge. Moreover, the proposed framework stands as one of the first automated SNOMED CT concept annotation systems in a Thai healthcare context. The framework has been systematically developed to showcase the performance on Thai medical documents in a manner that can be adaptable to other low-resourced languages and applicable to a wider range of clinical domains, enabling diverse use cases within clinical workflows; however, further development and evaluation are needed on specific use cases and goals.

### Limitations

Several limitations should be acknowledged when interpreting the study’s results. First, the generalizability of the findings is inherently constrained by the characteristics of the reference data. The annotated corpus was developed from only 220 clinical documents obtained from three departments within a single tertiary care teaching hospital. Although these documents represent diverse clinical narratives, they may not fully capture the linguistic diversity, localized dialects, or varied documentation shorthand used across Thailand’s health system. Consequently, the reported model performance should be interpreted as evidence of feasibility within similar clinical settings rather than as definitive performance across all Thai healthcare environments. Furthermore, our proposed proof-of-concept framework was developed and evaluated exclusively using Thai-English clinical text, its applicability beyond similar bilingual clinical settings remains unverified. Extension to other institutions, healthcare systems, or languages will require additional annotated datasets, local adaptation of the annotation guideline, and external validation to ensure robust performance.

Second, the construction of a ground truth dataset inevitably involved a degree of subjectivity. Although annotation and SNOMED CT mapping processes were performed by clinically trained informaticians and subsequently reviewed by an experienced clinician to maximize medical validity, interpretation of entity boundaries and selection among multiple clinically acceptable SNOMED CT concepts remained subject to expert judgment. Because annotation followed an iterative consensus process, formal inter-annotator agreement score was not assessed, as independent parallel annotations were not available. Annotation uncertainty may have influenced the reported performance metrics in several ways. Ambiguous entity boundaries, alternative but clinically acceptable SNOMED CT concepts, and borderline annotation decisions could result in model predictions being classified as FPs or FNs despite representing clinically reasonable interpretations. Consequently, evaluation metrics should be interpreted relative to the operational reference standard defined by the finalized annotation guideline rather than as an absolute measure of clinical correctness. To improve transparency and reproducibility, the final annotation guideline has been released as S1 Text, enabling future studies to reproduce or further refine the annotation process.

Third, the models deployed in this study were not trained on Thai data because of limited data availability and resource constraints. Both the embedding model (SapBERT [32]) and the re-ranking model (bge-large) require a substantial number of annotated mappings to finetune which are commonly lacking in low-resource language settings and are labor intensive to create. Although open-source LLMs can be further trained on available Thai medical documents, the computational resource requirements are huge. LLMs require a substantial financial investment for the deployment of closed-source models and significant computational resources for open-source models, which limits their practical implementation. Additionally, the use of Gemini 2.5 pro in the development process may introduce development bias favoring Gemini, as the prompts were tailored to its response characteristics.

Forth, the open-source LLMs (MedGemma and Qwen) were evaluated using the quantized versions (Q5_K_M and Q4_K_XL, respectively) because of limited computational resources. Although these quantization models require less memory and enable inference on commodity hardware, their performance may be modest compared with the corresponding full-precision models. Therefore, the reported performance metrics should be interpreted as conservative estimates. In addition, inference speed and computational resource utilization may vary depending on the hardware configuration and deployment environment, which may affect reproducibility across different systems.

Last, direct evaluation metric comparisons between this and previous studies should be interpreted with caution due to data heterogeneity. Variations in data distribution, annotation protocols, and patient characteristics may also influence reported performance. Despite these differences, the overall findings remain broadly comparable and indicative of general trends.

### Recommendation for Future Research

Future research should evaluate this proof-of-concept framework across multiple healthcare hospitals including hospitals of different sizes, care levels, and geographic regions to evaluate its external validity, scalability and generalizability. Additionally, expanding the annotated dataset to include more boarder range of clinical specialties, diverse populations, and document types, such as radiology reports, pathology reports, operative notes, discharge summary, and emergency notes, will improve the coverage of local relevant clinical concepts and provide a more comprehensive representation of Thai healthcare documentations. In addition, further refinement and validation of the annotation guideline across multiple annotators and institutions would strengthen the robustness and reproducibility of the reference dataset.

Adapting and validating the framework for other languages or multilingual clinical settings would help determine its applicability beyond the Thai-English context. Our study examines the feasibility of incorporating multiple readily available models to accomplish a chained task. This framework can be adopted and used in the context of low-resource language settings. Future studies could investigate whether fine-tuning these models using available local data can improve performance for specific use cases and settings.

To increase the practicality of the framework, investigating ways to reduce resource consumption is necessary. The smaller and context-specific NER models should be investigated to reduce resource consumption and increase the utility of this approach in resource-limited settings. Evaluating more open-source models could inform improved model options for implementation choices. Such tools may offer important opportunities to improve clinical data standardization, generate ICD-10 and ICD-11 codes, support cohort construction, and facilitate patient identification, particularly in settings where health information resources are limited. However, there is currently limited evidence regarding the real-world impact of these tools in low- and middle-income countries (LMICs). Their use could also amplify existing challenges, including non-standard abbreviations and jargon, typographical errors, contextual modifiers, and multilingual code-switching, potentially introducing systematic errors into clinical data, national health information systems, and downstream research infrastructure. Therefore, context-specific validation and assessment of both effectiveness and potential harm are needed before these tools are integrated into routine clinical or health-system workflows. Moreover, the potential benefits and harms of applying and accelerating the use of AI-based tools in LMICs should be carefully evaluated before widespread implementations. Such tools may offer important opportunities to improve clinical data, national health information system, and downstream research infrastructure. Therefore, context-specific validation and assessment of both effectiveness and potential harm are needed before these tools are integrated into routine clinical or health-system workflows.

The discrepancies and proximities of SNOMED CT concepts should be further considered, and a more standardized approach and practice for the use of these concepts could offer additional benefit for the implementation of this terminology. The culturally specific concepts should also be extended, especially herbal medicine, which is deeply rooted in Oriental cultures, thereby expanding the coverage and usability of SNOMED CT in Asian countries. This type of study could contribute to the improvement of the ontological structure and content of SNOMED CT and help guide alignment with other medical ontologies.

## Conclusion

The automatic concept extraction from Thai medical documents is now feasible due to advancements in LLMs. Performance is limited with open-source models, but closed-source solutions can successfully transform free-text into an internationally compliant standard, SNOMED CT. This study also provides domain-specific insights into how Thai linguistic structure, clinical phrasing, and documentation practices influence extraction and mapping outcomes. The error analysis reveals multiple failures that stem not only from the model’s inconsistent outputs but also from more complex linguistic factors and limitations of SNOMED CT with respect to local clinical expressions. This study highlights the urgent need for localized interface terminology and less labor-intensive evaluation strategies for multilingual clinical NLP by demonstrating the influences of these factors on the outputs generated. Resource consumption also persists as a major challenge, and further refinement is still required for use-case-specific implementation. Nevertheless, this automated approach can further enhance data quality, promote interoperability, and facilitate better utilization of healthcare data, contributing to improved patient care delivery and creating opportunities for broader international collaboration.

## Materials and methods

### Ethics statement

The study protocol was approved by the Ramathibodi Human Research Ethics Committee (Approval No.COA.MURA2023/469). Owing the retrospective study design, the requirement of informed consent was waived. Prior to annotation, all patient documents were assigned a study-specific identification number to replace original identifiers. Direct patient identifiers including names, hospital numbers, and visit date were removed. Potential person name within the free text portion of the data were further pseudo-anonymized by replacing with placeholders (e.g., <<name>> or <<doctor_name>>). Consequently, only pseudo-anonymized text was used for annotation and analysis, and no directly identifying information was intentionally retained or disclosed.

### Dataset

Given resource constraints, a total of 220 documents, which represented a feasible sample size for manual annotation, were retrieved retrospectively from electronic medical records at Chakri Naruebodindra Medical Institute, Ramathibodi Hospital, Mahidol University, covering the period from January 2019 to April 2023. Stratified cluster sampling was performed proportionally in line with the size of three independent departments: family medicine (n = 36), emergency (42), and internal medicine (142). Documents were categorized by ICD-10 chapter for the primary diagnosis, excluding chapter P (Perinatal disorders), chapter Q (Congenital and chromosomal abnormalities), and chapters V–Y (External causes of morbidity and mortality) for each department. The exclusion of perinatal and congenital disorders reflected the source population requirements for predominantly adult patient populations, while external causes were omitted as they typically serve as secondary diagnoses. Documents were randomly selected from each ICD-10 cluster, with care taken to ensure no duplicated retention of patients in the final dataset of 220 documents.

Of these 220 documents, 20 were randomly selected as a development set to create annotation guidelines and a working framework. The remaining 200 documents served as test set for evaluating the finalized framework and comparing LLM performance. The analysis focused specifically on the free-text narratives added to the chief complaint and present illness sections of the document.

### Dataset annotation

Iterative annotation was undertaken following the previously described methodology for the SNOMED CT entity challenge using the development dataset [11]. However, that approached considered only three domains: clinical findings, procedures, and anatomical structures. This study expanded the scope to also include five additional SNOMED CT domains: medication, laboratory, observable entity, event, and score. The resulting annotation guidelines outlined how text sequences should be annotated as entities along with domain-specific considerations. The final version of annotation guideline is provided as S1 Text.

Entity spans and their respective domains were annotated using Doccano [33], an open-source tool deployed on a local server. Each annotated entity was then mapped to SNOMED CT using Snap2SNOMED, an open-sourced mapping tool provided by SNOMED International. Annotation was conducted iteratively by two informatician annotators (NT and WP) according to the annotation guideline. Rather than performing independent duplicate annotation, both annotators continuously refined the annotation guideline during the annotation process. All annotated entities and SNOMED CT mappings were subsequently reviewed by an experienced physician (KS) using a custom-built web application developed by the research team (S1 Fig). Ambiguous entity boundaries, uncertain mappings, and disagreements were resolved through discussion until consensus was reached, and the agreed decisions were incorporated into subsequent revisions of the annotation guideline. The finalized guideline was then used to construct the reference dataset.

### Framework development

The framework was implemented using LangChain (version 0.3.27), a Python library for building LLM applications. The framework process was iteratively developed (i.e., prompt development, model experimentations, and variable adjustments) and evaluated in the development dataset using Gemini 2.5 Pro as the foundational model for prompt structures and logic refinement. All selected models were systemically evaluated using the testing dataset once all prompts were satisfactorily finalized.

The framework design consisted of three sequential components: NER, concept retrieval, and concept re-ranking. Each component was reliant on processing the output from the previous input as illustrated in Fig 2. The final output was a structured JavaScript Object Notation (JSON) representation of the source document with mapped SNOMED CT concept identifiers and corresponding metadata. All materials, framework design, prompts, and evaluation scripts are freely accessible from a repository available at: https://github.com/Chirurgo/LLM4SNOMED to support transparency, reproducibility and independent validation.

**Fig 2:**
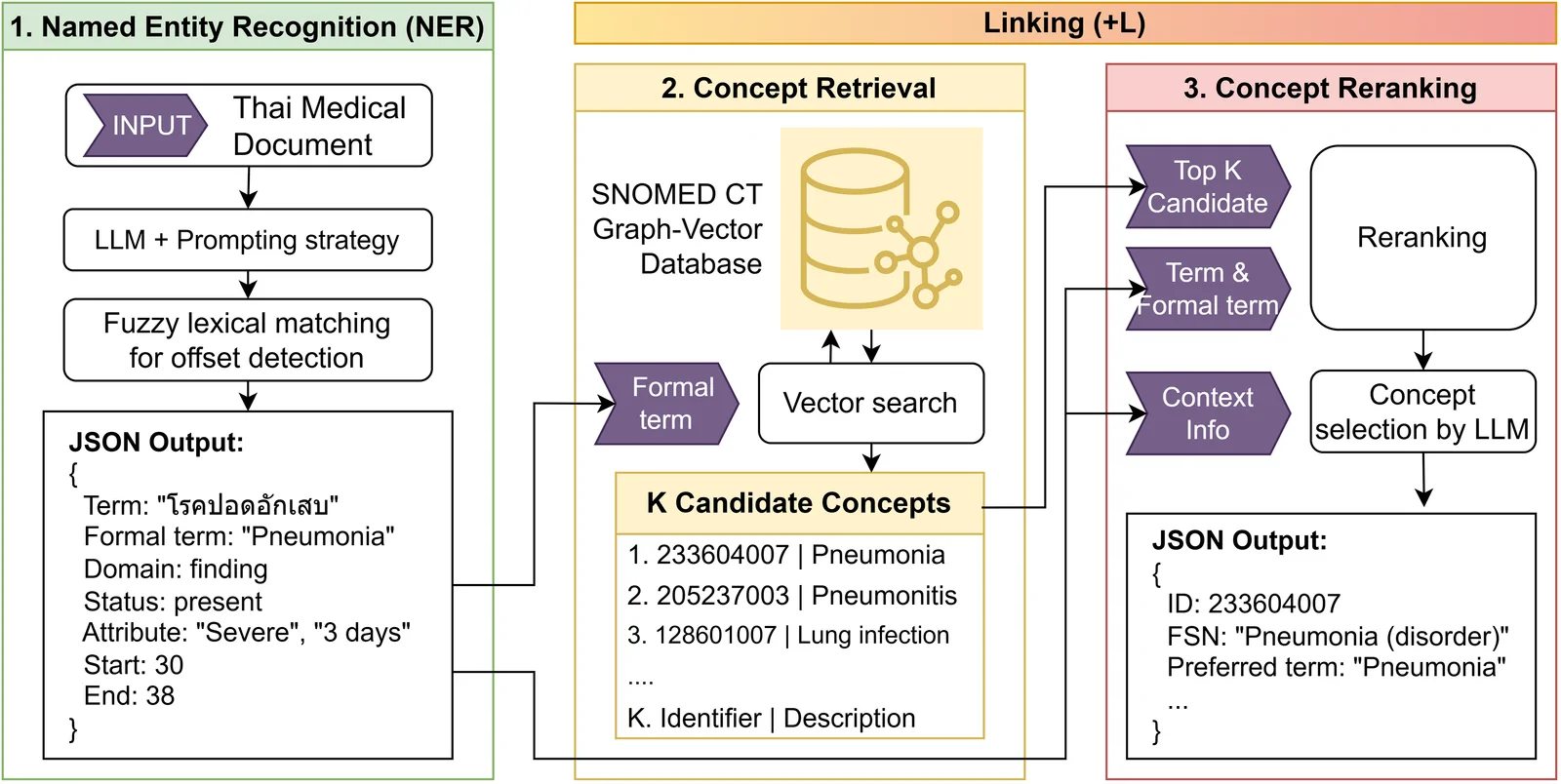
The high-level framework architecture. The framework consisted of three components where the outputs from each component formed the inputs (purple) for the next component. NER: named entity recognition, LLM: large language model, JSON: javascript object notation, ID: identifier, FSN: fully specified name.

#### Component 1: NER.

Extraction of clinical entities and contextual qualifiers from Thai clinical text was performed using a prompt-based LLM strategy. To ensure downstream compatibility, model outputs were strictly constrained to a predefined JSON schema consisting of 6 structured outputs: (1) the original entity form, (2) a normalized English rendering form, (3) one of eight predefined clinical domain labels, (4) character offsets, and two additional information to handle clinical negation and individual patient’s nuances: (5) pre-defined contextual status (e.g., present, absent, hypothetical, planned, etc.), (6) undefined key/value attributes where the model can decide the keys (e.g., dosage, laterality, measurement values, etc.). Character offsets were post-processed and aligned to the source text using a fuzzy lexical-matching given raw offset predictions by LLMs were considered unreliable. The prompt architecture was designed and underwent an iterative refinement process. The performances for each major improvement and the details of prompt refinement are provided in S3-S4 Tables. The final prompt architecture (see S5 Table) consisted of the following:

- Context: Setting the medical context and overall task.
- Task: Detailing the goal, entity domain descriptions, reasoning step, additional information (status, attributes), and strict output format.
- Example: A total of 4 records containing 66 example entities.

#### Component 2: Concept retrieval.

The normalized English rendering, outputted by prompt-based translation from component 1, served as the primary query to retrieve candidate SNOMED CT concepts for each extracted entity. Only SNOMED CT internation edition (version 2025-03-01) was used. Neither additional terminological nor local resource was added in consideration for the generalizability to other low-resource languages. SNOMED CT terminology was transformed into a Neo4j (v5.26) database compatible graphical representation encompassing concept and description nodes, and associated relationships to facilitate comprehensive querying. SNOMED CT descriptions included high-dimensional vector (embeddings) to enable semantic searching. Fig 3 illustrates this architecture, visualizing the “Infectious disease of lung (disorder)” concept (central, yellow), its relationships (arrow) to other concepts (yellow), and its associated description nodes (pink). The panel to the right shows the vector representation (embedding) of “Lung infection” description (pink circle with blue hue).

**Fig 3:**
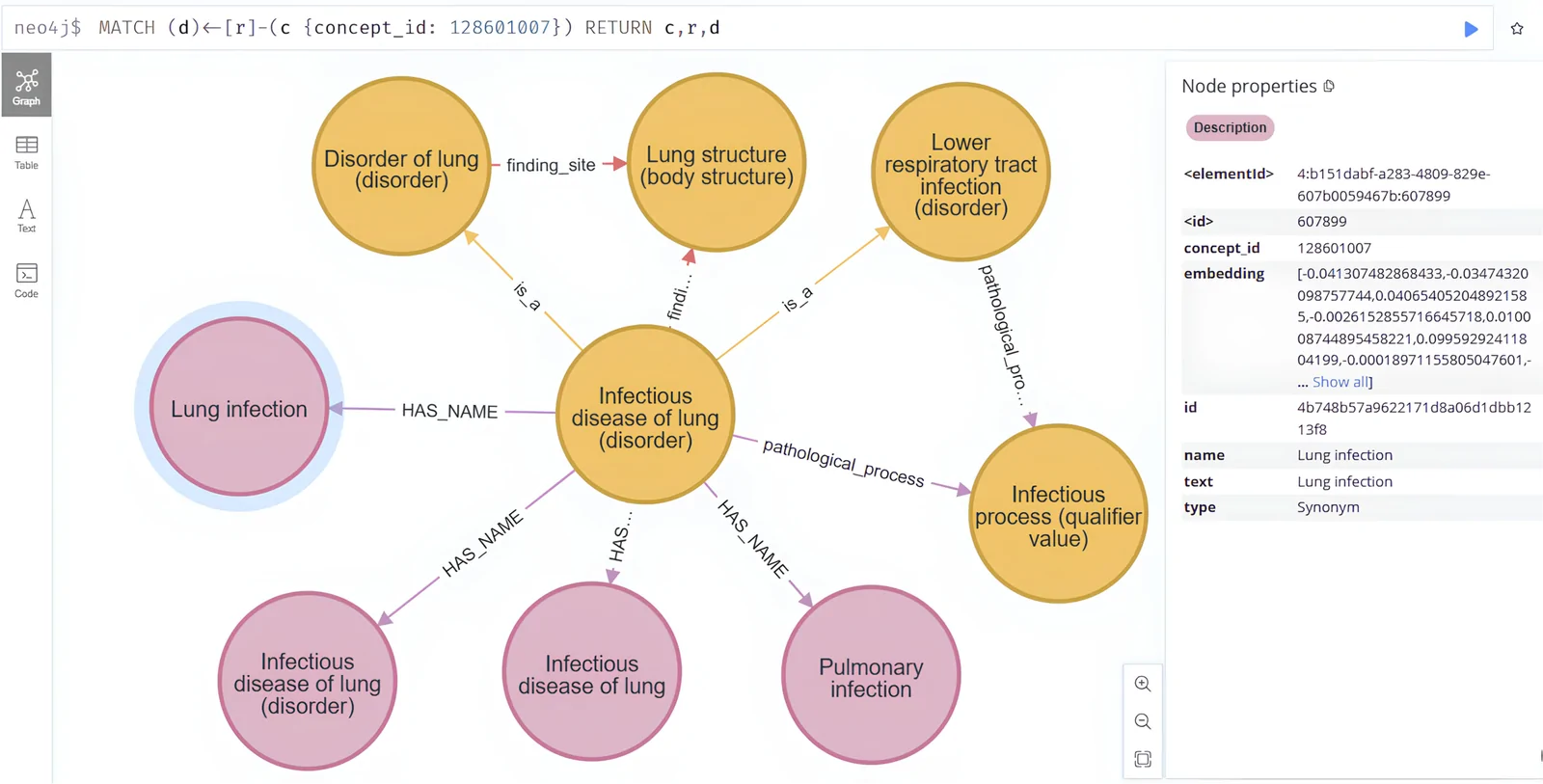
Example of a SNOMED CT concept “Infectious disease of lung (disorder)” in graph-vector representation.

Candidate concepts were retrieved via cosine similarity scores, returning a ranked Top-K candidate list for each entity. Several embedding models were benchmarked during the development phase; SapBERT [32] provided the best retrieval performance for the development dataset and was therefore selected as the foundational embedding model for all subsequent experiments. A comprehensive comparison of the embedding models and their respective retrieval metrics is provided in the S6-S7 Tables. The retrieval module returned a ranked list of candidate SNOMED CT concepts identifiers along with their matched preferred terms for each entity.

#### Component3: Concept reranking and final mapping.

The retrieved candidates were subsequently re-ranked to identify the most semantically appropriate SNOMED CT concept for each entity. Re-ranking was treated as a controlled tuning task, where multiple re-ranking models and the key parameters were systematically compared including: (1) the number of top-K candidates passed to the re-ranker; (2) re-ranking used the original Thai term versus the normalized English query; and (3) the amount of surrounding context included with the query (see S8-S9 Tables). Each configuration produced a single best concept assignment per entity, and mapped entities were consolidated into a single structured JSON output per source document.

The final system used bge-large as re-ranking model that was provided with a normalized English entity term and the 20 retrieved candidate terms without surrounding context. Number of retrieved candidates was evaluated, and the performance peaked at 20 candidates, see S8 Table. For the top-1 pipeline, the re-ranking module selected the highest-ranked SNOMED CT concept and provided its identifier with supporting metadata (e.g., fully specified name, semantic tag, and preferred terms).

For the LLM-selection pipeline, the preferred terms of the top 5 re-ranked candidates were presented to the LLM, which selected the single best-matching SNOMED CT concepts. The number of candidates presented to the LLM was determined based on top-k accuracy with 20 retrieved candidates: k = 1 (59.78%), k = 3 (76.91%), k = 5 (81.01), and k = 10 (85.29%), S8 Table. K = 5 was selected from the substantial performance gains from smaller k, while avoiding the need to present a large candidate set to the LLM. Finally, all outputs from three components were compiled into a document-level JSON structure containing the mapped entities.

### Evaluation and analysis

The LLMs were evaluated based on their multilingual processing capabilities constrained by funds available and computational resources. The closed-source models comprised GPT4.1 [34] and Gemini 2.5 Pro [35], both configured with a temperature of 0 to ensure deterministic outputs. The open-source models, Qwen 3 [36] (qwen3-30b-a3b-instruct-2507, Q5_K_M, 21.73 GB) and MedGemma [37] (medgemma-27b-it, Q4_K_XL, 18.51 GB) were deployed locally using LM studio (v0.3.30) with a fixed seed ensuring reproducibility. These open-source models were implemented on an NVIDIA RTX 3090 GPU, 12-core CPU and 128 GB of RAM. The human-annotated dataset represented the ground truth for model performance comparisons across each of the three aspects: (1) NER; (2) NER + L; and (3) Resource requirements and error analysis. The definitional examples of each evaluation metrics can be found in S10 Table.

#### NER.

NER is a multiclass sequence labeling task that can be evaluated using precision, recall, and F1 score. Identification of true positive (TP), FP, and FN were characterized as reported previously [26] according to two main criteria: (1) Strict criteria: A predicted entity was classified as a TP only if the predicted text span and clinical domain exactly matched the ground truth. (2) Loose criteria: A predicted entity was classified as a TP if there were a partial span overlap and an exact match in the clinical domain. For example, if “T2DM” is annotated as the gold entity, the model has to strictly predict the complete entity “T2DM” to classify as TP under strict matching criterion. If the model predicts only part of the entity (e.g., “DM”), the prediction is considered incorrect under the strict criterion but correct under loose criterion.

#### NER + L.

Two framework variations were evaluated: (1) Top-1 selection used the best result from BERT-based re-ranking model as the final mapping, and (2) LLM selection of the final mapping was chosen from the top five candidates from the re-ranking model, see S11 Table for the prompt developed for this task providing the task instruction, original textual form of the entity, normalized English form, and the top five candidates. The NER + L were evaluated by:

- Character-level IoU was used to compute the proportion of correctly identified character-level intersection for all concept mentions identified by either the ground truth or the model prediction (the union) without relying on predefined word boundaries. This was replicated from the SNOMED CT Entity Challenge [11]. Given a set of character from ground truth span G and predicted span P, the IoU is calculated as:

$$\begin{array}{c} \text{IoU}=\frac{\left|G\cap P\right|}{\left|G\cup P\right|} \end{array}$$

Where |·|denote the number of characters in the set. For example, if G= T2DM and P= DM, then G∩P = DM (2 characters) and G∪P = T2DM (4 characters), the IoU = 2/4 = 0.5 given the concept is exactly matched. This method provides a continuous evaluation that penalizes boundary inaccuracies proportionally, ensuring robustness across languages regardless of syntax complexity or explicit word delimiters. The mean IoU across all identified concepts for each document were subsequently averaged to obtain the final IoU score, see S12 Table.

- Strict entity-level evaluation modified those used for strict NER (i.e., precision, recall, and F1 score) but focused on the correctness of the linked concept identifier, using the SNOMED CT Concept ID as the reference label. The predicted text span and concept was required to exactly match the ground truth span and concept ID.
- Hierarchical-aware evaluation was introduced, accounting for detailed granularity of SNOMED CT. Loose criteria were modified to account for the granularity of SNOMED CT by leveraging the hierarchical structure of terminology. Under hierarchical-aware matching, a partial overlap of the text span, and the predicted concept is within a list of concepts directly related by one level of “is-a” relationship to the ground-truth concept. In summary, the predicted concept was considered correct if it was a child or parent concept of the ground truth concept.

#### Resource consumption and error analysis.

Resource consumption was documented including the average values per document which was calculated as total resource requirement divided by the total number of documents. Financial costs were calculated based on actual API expenditure for closed-source models. Indirect costs for open-source models (i.e., electric and hardware maintenance bills) were not explicitly measured or reported.

Error analysis was conducted using a custom web application (S1-S2 Fig). A total of 20 documents were randomly selected for manual review and detailed examination of all incorrectly extracted or linked entities. Errors were categorized as textual and linguistic factors, LLM interpretation errors, and SNOMED CT-related challenges.

## Supporting information

S1 Table Entity domain versus SNOMED CT semantic tag in the gold dataset. (DOCX)

S2 Table Distributions of Chapter level ICD-10 in gold dataset. (DOCX)

S3 Table NER performance of Gemini 2.5 for different prompting techniques on 5 documents from development dataset. (DOCX)

S4 Table NER performance of Gemini 2.5, GPT4.1, and Claude Sonnet 4 on 20 documents from development dataset. (DOCX)

S5 Table Prompt for NER. (DOCX)

S6 Table Top-K accuracy (%) results from different embedding models on entity extracted by three close-sourced LLMs. (DOCX)

S7 Table Top-K accuracy (%) results from reranking models. (DOCX)

S8 Table Top-K accuracy (%) of different number of candidates using bge-large reranking model and fixed context window size (40). (DOCX)

S9 Table Top-K accuracy (%) of different context window size using bge-large reranking model and fixed number of candidates (20). (DOCX)

S10 Table Definitional examples of each evaluation criteria and metrics. Example sentence: “DLP on simvas 10 1*1 วันนี้มากับลูก TC 223, ค่าLDL 135 HbA1C 5.8 FBS 96”, TP: true positive, FP: false positive, FN: false negative.
(DOCX)

S11 Table Prompt for LLM selection. (DOCX)

S12 Table Pseudo-code of Intersection over Union (IoU) calculation. cid: concept identification number, G: Gold entity, P: Predicted entity. (DOCX)

S1 Text Final version of the annotation guideline. (DOCX)

S1 Fig User Interface of the web application for error analysis build by researcher (A). (TIF)

S2 Fig User Interface of the web application for error analysis build by researcher (B). (TIF)

## Abbreviations

API: Application Programming Interface
FN: False Negative
FP: False Positive
JSON: JavaScript Object Notation
ICD-10: International Classification of Diseases, Tenth Revision
IoU: Intersection over Union
LLM: Large Language Model
NER: Named Entity Recognition
NER + L: Named Entity Recognition and Linking
NLP: Natural Language Processing
SNOMED CT: Systemized Nomenclature of Medicine – Clinical Terms
TP: True Positive
USD: United States Dollar
