# Supplementary material for: A framework for structuring Thai medical documents using systematized nomenclature of medicine clinical terms and large language models
Source: PLOS Digit Health. 2026 Oct 30;5(10):e0001791. doi: 10.1371/journal.pdig.0001791 (PMC13722323; doi:10.1371/journal.pdig.0001791)
Supplement: Supplementary file 1 [file pdig.0001791.s001.docx]

S1 Table. Entity domain versus SNOMED CT semantic tag in the gold dataset

| SNOMED CT semantic tag | anatomy | event | findings | laboratory | medication | observable entity | procedure | score | TOTAL |
| --- | --- | --- | --- | --- | --- | --- | --- | --- | --- |
| assessment scale | 0  (0.00) | 0  (0.00) | 0  (0.00) | 0  (0.00) | 0  (0.00) | 0  (0.00) | 0  (0.00) | 24  (0.38) | 24  (0.38) |
| body structure | 317  (4.96) | 0  (0.00) | 0  (0.00) | 0  (0.00) | 0  (0.00) | 0  (0.00) | 0  (0.00) | 0  (0.00) | 317  (4.96) |
| disorder | 0  (0.00) | 0  (0.00) | 1078  (16.87) | 0  (0.00) | 0  (0.00) | 0  (0.00) | 0  (0.00) | 0  (0.00) | 1078  (16.87) |
| event | 0  (0.00) | 49  (0.77) | 0  (0.00) | 0  (0.00) | 0  (0.00) | 0  (0.00) | 0  (0.00) | 0  (0.00) | 49  (0.77) |
| finding | 0  (0.00) | 0  (0.00) | 2,067  (32.35) | 0  (0.00) | 3  (0.05) | 1  (0.02) | 0  (0.00) | 0  (0.00) | 2,071  (32.41) |
| medicinal product | 0  (0.00) | 0  (0.00) | 0  (0.00) | 0  (0.00) | 93  (1.46) | 0  (0.00) | 0  (0.00) | 0  (0.00) | 93  (1.46) |
| medicinal product form | 0  (0.00) | 0  (0.00) | 0  (0.00) | 0  (0.00) | 4  (0.06) | 0  (0.00) | 0  (0.00) | 0  (0.00) | 4  (0.06) |
| morphologic abnormality | 0  (0.00) | 0  (0.00) | 75  (1.17) | 0  (0.00) | 0  (0.00) | 0  (0.00) | 0  (0.00) | 0  (0.00) | 75  (1.17) |
| observable entity | 0  (0.00) | 0  (0.00) | 0  (0.00) | 30  (0.47) | 0  (0.00) | 128  (2.00) | 0  (0.00) | 22  (0.34) | 180  (2.82) |
| organism | 0  (0.00) | 0  (0.00) | 0  (0.00) | 0  (0.00) | 1  (0.02) | 12  (0.19) | 0  (0.00) | 0  (0.00) | 13  (0.20) |
| physical force | 0  (0.00) | 1  (0.02) | 0  (0.00) | 0  (0.00) | 0  (0.00) | 0  (0.00) | 0  (0.00) | 0  (0.00) | 1  (0.02) |
| physical object | 0  (0.00) | 0  (0.00) | 0  (0.00) | 0  (0.00) | 0  (0.00) | 2  (0.03) | 0  (0.00) | 0  (0.00) | 2  (0.03) |
| procedure | 0  (0.00) | 0  (0.00) | 0  (0.00) | 1,025  (16.04) | 3  (0.05) | 0  (0.00) | 916  (14.33) | 0  (0.00) | 1,944  (30.42) |
| product | 0  (0.00) | 0  (0.00) | 0  (0.00) | 0  (0.00) | 14  (0.22) | 0  (0.00) | 0  (0.00) | 0  (0.00) | 14  (0.22) |
| regime/therapy | 0  (0.00) | 0  (0.00) | 0  (0.00) | 0  (0.00) | 7  (0.11) | 0  (0.00) | 81  (1.27) | 0  (0.00) | 88  (1.38) |
| situation | 0  (0.00) | 0  (0.00) | 30  (0.47) | 0  (0.00) | 0  (0.00) | 0  (0.00) | 8  (0.13) | 0  (0.00) | 38  (0.59) |
| substance | 0  (0.00) | 0  (0.00) | 0  (0.00) | 0  (0.00) | 399  (6.24) | 0  (0.00) | 0  (0.00) | 0  (0.00) | 399  (6.24) |
| TOTAL | 317  (4.96) | 50  (0.78) | 3,250  (50.86) | 1,055  (16.51) | 524  (8.20) | 143  (2.24) | 1,005  (15.73) | 46  (0.72) | 6,390  (100.00) |
