# Supplementary material for: A framework for structuring Thai medical documents using systematized nomenclature of medicine clinical terms and large language models
Source: PLOS Digit Health. 2026 Oct 30;5(10):e0001791. doi: 10.1371/journal.pdig.0001791 (PMC13722323; doi:10.1371/journal.pdig.0001791)
Supplement: Supplementary file 2 [file pdig.0001791.s002.docx]

S2 Table. Distributions of Chapter level ICD-10 in gold dataset

| ICD Chapter | Sample (%) | ICD Chapter | Sample (%) |
| --- | --- | --- | --- |
| A | 10 (4.55) | O | 1 (0.45) |
| B | 3 (1.36) | P | 0 (0.00) |
| C | 2 (0.91) | Q | 0 (0.00) |
| D | 3 (1.36) | R | 32 (14.55) |
| E | 31 (14.09) | S | 5 (2.27) |
| F | 1 (0.45) | T | 1 (0.45) |
| G | 6 (2.73) | U | 2 (0.91) |
| H | 2 (0.91) | V | 0 (0.00) |
| I | 37 (16.82) | W | 0 (0.00) |
| J | 13 (5.91) | X | 0 (0.00) |
| K | 24 (10.91) | Y | 0 (0.00) |
| L | 3 (1.36) | Z | 19 (8.64) |
| M | 18 (8.18) | No ICD | 0 (0.00) |
| N | 7 (3.18) | TOTAL | 220 (100.00) |
