# Supplementary material for: A framework for structuring Thai medical documents using systematized nomenclature of medicine clinical terms and large language models
Source: PLOS Digit Health. 2026 Oct 30;5(10):e0001791. doi: 10.1371/journal.pdig.0001791 (PMC13722323; doi:10.1371/journal.pdig.0001791)
Supplement: Supplementary file 3 [file pdig.0001791.s003.docx]

S3 Table. NER performance of Gemini 2.5 for different prompting techniques on 5 documents from development dataset

| Prompting technique | Strict criteria (%) | | | Loose criteria (%) | | |
| --- | --- | --- | --- | --- | --- | --- |
|  | Precision | Recall | F1 score | Precision | Recall | F1 score |
| Zero-shot and structured output | 69.41 | 54.18 | 60.86 | 81.83 | 63.88 | 71.75 |
| Few-shot with examples (3 documents, 32 entities) | 68.32 | 63.52 | 65.83 | 80.83 | 75.15 | 77.89 |
| Thinking and reasoning with added field “reasoning_steps” | 75.00 | 66.18 | 70.31 | 84.17 | 74.26 | 78.91 |
| Context extraction by adding fields “status” and “attribute” | 82.20 | 71.32 | 76.38 | 88.98 | 77.21 | 82.68 |
| Adjust and add more examples (4 documents, 66 entities) | **85.47** | **75.77** | **80.33** | **89.53** | **79.38** | **84.15** |
