# Supplementary material for: A framework for structuring Thai medical documents using systematized nomenclature of medicine clinical terms and large language models
Source: PLOS Digit Health. 2026 Oct 30;5(10):e0001791. doi: 10.1371/journal.pdig.0001791 (PMC13722323; doi:10.1371/journal.pdig.0001791)
Supplement: Supplementary file 4 [file pdig.0001791.s004.docx]

S4 Table. NER performance of Gemini 2.5, GPT4.1, and Claude Sonnet 4 on 20 documents from development dataset

| Model | Number of found entity | Strict criteria (%) | | | Loose criteria (%) | | |
| --- | --- | --- | --- | --- | --- | --- | --- |
|  |  | Precision | Recall | F1 score | Precision | Recall | F1 score |
| Gemini 2.5 | **522** | 79.29 | **70.81** | **74.81** | 88.19 | 78.76 | **83.21** |
| GPT4.1 | 516 | 74.89 | 70.66 | 72.71 | 84.69 | **79.91** | 82.23 |
| Claude Sonnet 4 | 469 | **81.65** | 64.31 | 71.95 | **90.28** | 71.10 | 79.55 |
