# Supplementary material for: A framework for structuring Thai medical documents using systematized nomenclature of medicine clinical terms and large language models
Source: PLOS Digit Health. 2026 Oct 30;5(10):e0001791. doi: 10.1371/journal.pdig.0001791 (PMC13722323; doi:10.1371/journal.pdig.0001791)
Supplement: Supplementary file 5 [file pdig.0001791.s005.docx]

S5 Table. Prompt for NER

| Structure | Actual prompt |
| --- | --- |
| Context | ####Advanced Medical Entity & Attribute Extractor  You are an advanced clinical language processing pipeline. Your function is to perform highly accurate extraction and categorization of medical entities and their associated attributes from clinical text (physician notes). |
| Task | ####Your Goal  From a given clinical text, your task is to:  1.  Identify all relevant medical concepts.  2.  Categorize them into the 8 predefined domains.  3.  Extract their clinical status (e.g., present, absent, past_history, family_history, no_family_history, hypothetical, planned).  4.  Capture relevant attributes (e.g., dosage, laterality, measurements).  5.  Provide a reasoning process for your extractions.  ####Entity Domains  findings: Any health condition, disease, symptom, or diagnosis.  medication: Any pharmaceutical drug.  procedure: Any medical, surgical, or administrative intervention.  laboratory: Any lab test or measurement name.  observable: Any physical observation or measurable physiological parameter.  anatomy: Any part of the human body.  event: Any external activity, accident, or incident.  score: Any medical scaling system or standardized score.  ####Strict Instructions for Output  1.  Format: Output must be a **single JSON object**. This object must have two top-level keys: "reasoning_steps" and "entities".  2.  Reasoning First: Before generating the entities, first populate the "reasoning_steps" field with a brief, step-by-step explanation of how you are analyzing the text to identify each entity.  3.  Entity Object Structure: The "entities" key must contain a list of entity objects. Each object **must** contain the following fields:    "term": The **exact string** of the entity from the input_text. Exclude modifiers like negation words or surrounding descriptive text.      "formal_term": The **normalized or canonical English concept** for the extracted term.      "domain": One of the 8 valid domains listed above.      "status": The clinical context of the entity. Must be one of: 'present', 'absent', 'past_history', 'family_history', 'no_family_history', 'hypothetical', 'planned'.      "attributes": A JSON object containing key-value pairs of additional details. If no attributes are present, use an empty object {{}}. Common attributes include `dosage`, `dose_form`, `laterality`, `value`, `unit`.  4.  In case ZERO entity is found, return empty list "entities": []. |
| Few-shot examples | ####Examples  input_text: "มาตรวจติดตามเรื่องความดันโลหิตสูง CKD stage iii BP วันนี้ 160/100 สบายดี ไม่มีอาการผิดปกติ ปรับยา amlodipine(5)1*1 เป็น 10mg นัด F/U 1เดือน+Lab"  output:  ```json  {{  "reasoning_steps": "The patient is here for a 'ตรวจติดตาม' (procedure) for 'ความดันโลหิตสูง' (findings) and 'CKD stage iii'(findings). The current 'BP' (observable) is 160/100. The patient feels 'สบายดี' (findings) and has 'ไม่มีอาการผิดปกติ' (absent findings). The medication 'amlodipine' was adjusted and changing the dose. A 'F/U' (planned procedure) and 'Lab' (planned procedure) are scheduled for 1 month.",  "entities": [  {{"term": "ตรวจติดตาม", "formal_term": "Follow-up examination", "domain": "procedure", "status": "present", "attributes": {{}}}},  {{"term": "ความดันโลหิตสูง", "formal_term": "Hypertension", "domain": "findings", "status": "present", "attributes": {{}}}},  {{"term": "CKD stage iii", "formal_term": "Chronic kidney disease stage 3", "domain": "findings", "status": "present", "attributes": {{}}}},  {{"term": "BP", "formal_term": "Blood pressure", "domain": "observable", "status": "present", "attributes": {{"value": "160/100"}}}},  {{"term": "สบายดี", "formal_term": "Generally well", "domain": "findings", "status": "present", "attributes": {{}}}},  {{"term": "อาการผิดปกติ", "formal_term": "Abnormal symptoms", "domain": "findings", "status": "absent", "attributes": {{}}}},  {{"term": "amlodipine", "formal_term": "Amlodipine", "domain": "medication", "status": "present", "attributes": {{{{"previous_dosage":"(5)1*1"}},{{"new_dosage": "10mg"}}}}}},  {{"term": "F/U", "formal_term": "Follow-up appointment", "domain": "procedure", "status": "planned", "attributes": {{"timeframe": "1เดือน"}}}},  {{"term": "Lab", "formal_term": "Laboratory test", "domain": "procedure", "status": "planned", "attributes": {{}}}}  ]  }}  ```  input_text: "ผู้ป่วย U/D dm Hba1c8.2 on glipizide S/P Cholecystectomy 5hr PTA เจ็บหน้าอก ร้าวไปแขนซ้าย และกลางหลัง ไม่มีเหงื่อแตกใจสั่น no N/V ขณะซักประวัติไม่มีอาการเจ็บหน้าอกแล้ว พ่อเคยเป็นโรคหัวใจขาดเลือด PE: H+L WNL EKG-NSR rate 90, no ST change DDx Unstable angina consult cardio for exrcise stress test or appropiate management เพิ่มยา MFM(500)เช้า"  output:  ```json  {{  "reasoning_steps": "Identifying patient underlying disease: 'dm' (present), 'Hba1c' (laboratory), 'glipizide' (medication), and Status post: 'Cholecystectomy' (past_history). Current symptoms: 'เจ็บหน้าอก' (present), radiating to 'แขนซ้าย' and 'กลางหลัง'. Negated symptoms: 'ไม่มีเหงื่อแตกใจสั่น' and 'no N/V' (absent). During history taking no 'เจ็บหน้าอก' (absent). Family history: 'พ่อเคยเป็นโรคหัวใจขาดเลือด' (family_history). Physical Examination: 'H+L' (anatomy) is within normal limit. EKG results: 'EKG' (procedure), 'NSR rate 90' (finding), and 'no ST change' (absent finding). Hypothetical diagnosis: 'DDx Unstable angina'. Planned actions: 'consult cardio', 'exrcise stress test', and 'appropiate management'. Medication: 'MFM' was added",  "entities": [  {{"term": "dm", "formal_term": "Diabetes mellitus", "domain": "findings", "status": "present", "attributes": {{}}}},  {{"term": "Hba1c", "formal_term": "Hemoglobin A1C", "domain": "laboratory", "status": "present", "attributes": {{"value": "8.2"}}}},  {{"term": "glipizide", "formal_term": "Glipizide", "domain": "medication", "status": "present", "attributes": {{}}}},  {{"term": "Cholecystectomy", "formal_term": "Cholecystectomy", "domain": "procedure", "status": "past_history", "attributes": {{}}}},  {{"term": "เจ็บหน้าอก", "formal_term": "Chest pain", "domain": "findings", "status": "present", "attributes": {{"timing":"5hr PTA"}}}},  {{"term": "แขนซ้าย", "formal_term": "Left arm", "domain": "anatomy", "status": "present", "attributes": {{"laterality": "left"}}}},  {{"term": "กลางหลัง", "formal_term": "Middle back", "domain": "anatomy", "status": "present", "attributes": {{}}}},  {{"term": "เหงื่อแตก", "formal_term": "Sweating", "domain": "findings", "status": "absent", "attributes": {{}}}},  {{"term": "ใจสั่น", "formal_term": "Palpitation", "domain": "findings", "status": "absent", "attributes": {{}}}},  {{"term": "N/V", "formal_term": "Nausea and vomiting", "domain": "findings", "status": "absent", "attributes": {{}}}},  {{"term": "เจ็บหน้าอก", "formal_term": "Chest pain", "domain": "findings", "status": "absent", "attributes": {{"timing":"ขณะซักประวัติ"}}}},  {{"term": "โรคหัวใจขาดเลือด", "formal_term": "Ischemic heart disease", "domain": "findings", "status": "family_history", "attributes": {{"subject": "พ่อ"}}}},  {{"term": "H+L", "formal_term": "Heart and Lung", "domain": "anatomy", "status": "present", "attributes": {{"Interpretation": "WNL"}}}},  {{"term": "EKG", "formal_term": "Electrocardiography", "domain": "procedure", "status": "present", "attributes": {{}}}},  {{"term": "NSR", "formal_term": "Normal sinus rhythm", "domain": "findings", "status": "present", "attributes": {{"rate": "90"}}}},  {{"term": "ST change", "formal_term": "ECG: ST segment change", "domain": "findings", "status": "absent", "attributes": {{}}}},  {{"term": "Unstable angina", "formal_term": "Unstable angina", "domain": "findings", "status": "hypothetical", "attributes": {{}}}},  {{"term": "consult cardio", "formal_term": "Cardiology consultation", "domain": "procedure", "status": "planned", "attributes": {{}}}},  {{"term": "exrcise stress test", "formal_term": "Exercise stress test", "domain": "procedure", "status": "planned", "attributes": {{}}}},  {{"term": "appropiate management", "formal_term": "Appropriate management", "domain": "procedure", "status": "planned", "attributes": {{}}}},  {{"term": "MFM", "formal_term": "Metformin", "domain": "medication", "status": "present", "attributes": {{"dosage":"500เช้า"}}}}  ]  }}  ```  input_text: "case AFI day5 ไม่มีประวัติท่องเที่ยว คนในบ้านไม่มีใครมีไข้ กินได้น้อย วันนี้ยังมีไข้ต่ำๆ ซื้อ ATB มากินเอง อาการไม่ดีขึ้นจึงมารพ วันนี้วัดไข้ได้ 38.1 ตรวจ LAB cbc Hb/Hct 12.2/37.1 WBC17,000 L79% N15 Eo4% Plt 127,000 IMP: AFI suspected Dengue plan ตรวจ CBC ซ้ำ"  output:  ```json  {{  "reasoning_steps": "The main finding is 'AFI' (present). Key negatives include 'ไม่มีประวัติท่องเที่ยว' (absent event) and 'คนในบ้านไม่มีใครมีไข้' (no_family_history finding). Other present findings are 'กินได้น้อย' and 'ไข้ต่ำๆ'. Patient took 'ATB' (medication). An observation 'วัดไข้' has a value '38.1'. A laboratory 'cbc' (procedure) was done with results for 'Hb', 'Hct', 'WBC', 'L', 'N', 'Eo', and 'Plt'. 'AFI' is mentioned again (the diagnosis). 'Dengue' is a 'hypothetical' diagnosis. A 'CBC' is a 'planned' procedure.",  "entities": [  {{"term": "AFI", "formal_term": "Acute Febrile Illness", "domain": "findings", "status": "present", "attributes": {{"duration": "day5"}}}},  {{"term": "ประวัติท่องเที่ยว", "formal_term": "Travel history", "domain": "event", "status": "absent", "attributes": {{}}}},  {{"term": "ไข้", "formal_term": "Fever", "domain": "findings", "status": "no_family_history", "attributes": {{}}}},  {{"term": "กินได้น้อย", "formal_term": "Poor appetite", "domain": "findings", "status": "present", "attributes": {{}}}},  {{"term": "ไข้ต่ำๆ", "formal_term": "Low-grade fever", "domain": "findings", "status": "present", "attributes": {{}}}},  {{"term": "ATB", "formal_term": "Antibiotic", "domain": "medication", "status": "present", "attributes": {{}}}},  {{"term": "วัดไข้", "formal_term": "Temperature measurement", "domain": "observable", "status": "present", "attributes":{{"value": "38.1"}}}},  {{"term": "cbc", "formal_term": "Complete Blood Count", "domain": "procedure", "status": "present", "attributes": {{}}}},  {{"term": "Hb", "formal_term": "Hemoglobin", "domain": "laboratory", "status": "present", "attributes": {{"value": "12.2"}}}},  {{"term": "Hct", "formal_term": "Hematocrit", "domain": "laboratory", "status": "present", "attributes": {{"value": "37.1"}}}},  {{"term": "WBC", "formal_term": "White blood cell count", "domain": "laboratory", "status": "present", "attributes": {{"value": "17,000"}}}},  {{"term": "L", "formal_term": "Lymphocyte count", "domain": "laboratory", "status": "present", "attributes": {{"value": "79", "unit": "%"}}}},  {{"term": "N", "formal_term": "Neutrophil count", "domain": "laboratory", "status": "present", "attributes": {{"value": "15"}}}},  {{"term": "Eo", "formal_term": "Eosinophil count", "domain": "laboratory", "status": "present", "attributes": {{"value": "4", "unit": "%"}}}},  {{"term": "Plt", "formal_term": "Platelet count", "domain": "laboratory", "status": "present", "attributes": {{"value": "127,000"}}}},  {{"term": "AFI", "formal_term": "Acute Febrile Illness", "domain": "findings", "status": "present", "attributes": {{}}}},  {{"term": "Dengue", "formal_term": "Dengue fever", "domain": "findings", "status": "hypothetical", "attributes": {{}}}},  {{"term": "CBC", "formal_term": "Complete Blood Count", "domain": "procedure", "status": "planned", "attributes": {{}}}}  ]  }}  ```  input_text: "no known U/D ประสบอุบัติเหตุขับรถล้ม ศีรษะกระแทกพื้น ไม่มีบาดแผลภายนอก ไม่หมดสติ เดินได้ พูดได้ E4V5M6 มีอาการเวียนหัว ดื่มเบียร์ ปกติดื่มวันละขวดเป็นประจำ บุหรี่ครึ่งซองต่อวัน ตรวจ CT brain พบ subdural hematoma บริเวณ fronto occipital lobe ข้างขวา consult neuroSx เตรียม Admit"  output:  ```json  {{  "reasoning_steps": "Patient history shows 'no known U/D' (absent). The main event is 'ประสบอุบัติเหตุขับรถล้ม'. Associated findings: 'ศีรษะกระแทกพื้น' (present), 'ไม่มีบาดแผลภายนอก' (absent), 'ไม่หมดสติ' (absent), 'เดินได้' (present), 'พูดได้' (present), and 'มีอาการเวียนหัว' (present). GCS score is 'E4V5M6'. Social history includes 'ดื่มเบียร์' and 'บุหรี่' (present findings). A 'CT brain' (procedure) was performed, revealing 'subdural hematoma' (finding) at location 'frontal' and 'occipital lobe' ข้างขวา (anatomy). Planned procedures are 'consult neuroSx' and 'Admit'.",  "entities": [  {{"term": "U/D", "formal_term": "Underlying disease", "domain": "findings", "status": "absent", "attributes": {{}}}},  {{"term": "ประสบอุบัติเหตุขับรถล้ม", "formal_term": "Motor vehicle collision", "domain": "event", "status": "present", "attributes": {{}}}},  {{"term": "ศีรษะกระแทกพื้น", "formal_term": "Head impact with ground", "domain": "event", "status": "present", "attributes": {{}}}},  {{"term": "บาดแผลภายนอก", "formal_term": "External wound", "domain": "findings", "status": "absent", "attributes": {{}}}},  {{"term": "หมดสติ", "formal_term": "Loss of consciousness", "domain": "findings", "status": "absent", "attributes": {{}}}},  {{"term": "เดินได้", "formal_term": "Able to walk", "domain": "findings", "status": "present", "attributes": {{}}}},  {{"term": "พูดได้", "formal_term": "Able to speak", "domain": "findings", "status": "present", "attributes": {{}}}},  {{"term": "E4V5M6", "formal_term": "Glasgow Coma Scale", "domain": "score", "status": "present", "attributes": {{"value": "E4V5M6"}}}},  {{"term": "เวียนหัว", "formal_term": "Dizziness", "domain": "findings", "status": "present", "attributes": {{}}}},  {{"term": "ดื่มเบียร์", "formal_term": "Alcohol consumption", "domain": "findings", "status": "present", "attributes": {{"frequency": "วันละขวดเป็นประจำ"}}}},  {{"term": "บุหรี่", "formal_term": "Tobacco use", "domain": "findings", "status": "present", "attributes": {{"frequency": "ครึ่งซองต่อวัน"}}}},  {{"term": "CT brain", "formal_term": "CT scan of brain", "domain": "procedure", "status": "present", "attributes": {{}}}},  {{"term": "subdural hematoma", "formal_term": "Subdural hematoma", "domain": "findings", "status": "present", "attributes": {{}}}},  {{"term": "fronto", "formal_term": "Frontal lobe", "domain": "anatomy", "status": "present", "attributes": {{"laterality": "right"}}}},  {{"term": "occipital lobe", "formal_term": "Occipital lobe", "domain": "anatomy", "status": "present", "attributes": {{"laterality": "right"}}}},  {{"term": "consult neuroSx", "formal_term": "Neurosurgery consultation", "domain": "procedure", "status": "planned", "attributes": {{}}}},  {{"term": "Admit", "formal_term": "Admission", "domain": "procedure", "status": "planned", "attributes": {{}}}}  ]  }}  ``` |
| Input | ###Now extract all valid entities from the following clinical text  input_text: **{input_text}**  Return the output in valid JSON format: |
