# Supplementary material for: A framework for structuring Thai medical documents using systematized nomenclature of medicine clinical terms and large language models
Source: PLOS Digit Health. 2026 Oct 30;5(10):e0001791. doi: 10.1371/journal.pdig.0001791 (PMC13722323; doi:10.1371/journal.pdig.0001791)
Supplement: Supplementary file 6 [file pdig.0001791.s006.docx]

S6 Table. Top-K accuracy (%) results from different embedding models on entity extracted by three close-sourced LLMs

| Model | Model size | Vector dimension | Gemini2.5 pro 06-05  (n=522) | | | Gpt-4.1-2025-04-14  (n=516) | | | Claude-sonnet-4-20250514  (n=469) | | |
| --- | --- | --- | --- | --- | --- | --- | --- | --- | --- | --- | --- |
|  |  |  | K = 1 | K = 5 | K= 10 | K = 1 | K = 5 | K= 10 | K = 1 | K = 5 | K= 10 |
| text-embedding-ada-002 (OpenAI) | N/A | 1536 | 57.09 | 69.54 | 74.14 | 55.23 | 67.05 | 74.22 | 59.28 | 71.22 | 76.97 |
| GTE-mlm-base | 306M | 768 | 42.72 | 45.98 | 46.93 | 43.22 | 46.12 | 46.90 | 47.55 | 50.75 | 51.60 |
| PubmedBERT | 109M | 768 | 48.47 | 52.87 | 56.32 | 47.67 | 51.74 | 55.81 | 51.39 | 56.50 | 60.34 |
| BioClinicalBERT | 150M | 768 | 49.81 | 54.21 | 58.43 | 48.06 | 53.68 | 58.33 | 53.73 | 58.42 | 62.90 |
| GatorTron | 345M | 768 | 43.68 | 49.81 | 51.15 | 41.28 | 48.84 | 50.97 | 46.91 | 54.80 | 55.86 |
| SapBERT-UMLS | 278M | 768 | 56.32 | 69.35 | 74.14 | 55.04 | 65.50 | 68.60 | 57.14 | 70.58 | 74.41 |
| SapBERT-Pubmed  (CLS) | 109M | 768 | 59.00 | 73.75 | 79.50 | 56.20 | 68.80 | 73.84 | 61.62 | 76.12 | 82.30 |
| SapBERT-Pubmed  (Mean pooling) | 109M | 768 | 56.32 | 71.65 | 78.16 | 54.26 | 68.41 | 73.64 | 58.42 | 74.84 | 81.66 |
