# Supplementary material for: A framework for structuring Thai medical documents using systematized nomenclature of medicine clinical terms and large language models
Source: PLOS Digit Health. 2026 Oct 30;5(10):e0001791. doi: 10.1371/journal.pdig.0001791 (PMC13722323; doi:10.1371/journal.pdig.0001791)
Supplement: Supplementary file 7 [file pdig.0001791.s007.docx]

S7 Table. Top-K accuracy (%) results from reranking models

| Models | Size | Domain | Language | Top-K accuracy (%) | | |
| --- | --- | --- | --- | --- | --- | --- |
|  |  |  |  | K = 1 | K = 5 | K = 10 |
| No reranking | Baseline | - | - | 59.89 | 73.45 | 79.10 |
| Using original term | | | | | | |
| bge-large | 335M | General | Multilingual | 47.41 | 77.96 | 84.81 |
| jina-reranker-v2 | 278M | General | Multilingual | 45.96 | 71.63 | 84.20 |
| ms-marco-MiniLM-L6-v2 | 22.7M | General | English | 38.31 | 72.93 | 84.53 |
| MedCPT | 109M | Medical | English | 27.87 | 70.13 | 83.97 |
| PubmedBERT | 109M | Medical | English | 16.45 | 61.06 | 82.27 |
| BioClinicalBERT | 150M | Medical | English | 11.05 | 59.85 | 83.98 |
| Using translated term | | | | | | |
| bge-large | 335M | General | Multilingual | **59.78** | **81.01** | **85.29** |
| jina-reranker-v2 | 278M | General | Multilingual | 54.24 | 77.21 | 83.99 |
| ms-marco-MiniLM-L6-v2 | 22.7M | General | English | 44.81 | 76.14 | 82.70 |
| MedCPT | 109M | Medical | English | 26.94 | 73.99 | 84.32 |
| PubmedBERT | 109M | Medical | English | 15.41 | 54.13 | 82.02 |
| BioClinicalBERT | 150M | Medical | English | 19.96 | 66.79 | 82.21 |
