# Supplementary material for: A framework for structuring Thai medical documents using systematized nomenclature of medicine clinical terms and large language models
Source: PLOS Digit Health. 2026 Oct 30;5(10):e0001791. doi: 10.1371/journal.pdig.0001791 (PMC13722323; doi:10.1371/journal.pdig.0001791)
Supplement: Supplementary file 8 [file pdig.0001791.s008.docx]

S8 Table. Top-K accuracy (%) of different number of candidates using bge-large reranking model and fixed context window size (40)

| Number of candidates | Model selection | K = 1 | K = 3 | K = 5 | K = 10 |
| --- | --- | --- | --- | --- | --- |
| 5 | 50.18 | 49.64 | 64.73 | 65.27 | 65.27 |
| 10 | 55.35 | 57.35 | 73.87 | 76.59 | 77.50 |
| 15 | 54.28 | 56.47 | 73.95 | 77.41 | 79.60 |
| 20 | **56.80** | **59.78** | **76.91** | **81.01** | **85.29** |
| 30 | 55.17 | 58.08 | 72.96 | 77.86 | 82.58 |
| 50 | 54.50 | 58.17 | 75.05 | 77.98 | 82.94 |
| 100 | 56.25 | 56.99 | 72.06 | 77.21 | 82.17 |
