# Supplementary material for: A framework for structuring Thai medical documents using systematized nomenclature of medicine clinical terms and large language models
Source: PLOS Digit Health. 2026 Oct 30;5(10):e0001791. doi: 10.1371/journal.pdig.0001791 (PMC13722323; doi:10.1371/journal.pdig.0001791)
Supplement: Supplementary file 9 [file pdig.0001791.s009.docx]

S9 Table. Top-K accuracy (%) of different context window size using bge-large reranking model and fixed number of candidates (20)

| Context window size | Model selection | K = 1 | K = 5 | K = 10 |
| --- | --- | --- | --- | --- |
| 0 | **57.48** | **58.94** | **80.11** | **84.31** |
| 20 | 54.17 | 58.70 | 80.07 | 84.42 |
| 40 | 54.90 | 58.04 | 78.74 | 83.36 |
| 60 | 55.58 | 58.68 | 80.07 | 83.55 |
| 80 | 54.77 | 56.76 | 78.56 | 82.88 |
