# Supplementary material for: A framework for structuring Thai medical documents using systematized nomenclature of medicine clinical terms and large language models
Source: PLOS Digit Health. 2026 Oct 30;5(10):e0001791. doi: 10.1371/journal.pdig.0001791 (PMC13722323; doi:10.1371/journal.pdig.0001791)
Supplement: Supplementary file 10 [file pdig.0001791.s010.docx]

S10 Table. Definitional examples of each evaluation criteria and metrics

| Gold entity | | | | Predicted entity | | | | Domain level evaluations (NER) | | Concept level evaluations (NER+L) | | |
| --- | --- | --- | --- | --- | --- | --- | --- | --- | --- | --- | --- | --- |
| Text | Offset | Domain | Concept id | Text | Offset | Domain | Concept id | Strict | Loose | IoU | Strict | Hierarchical-aware |
| DLP | 1-3 | Findings | 370992007 | DLP | 1-3 | Findings | 370992007 | TP | TP | 1.0 (3/3) | TP | TP |
| simvas | 8-13 | Medication |  | - | - | - | - | FN | FN | 0.0 | FN | FN |
| - | - | - | - | วันนี้ | 22-27 | Observable | 406543005 | FP | FP | 0.0 | FP | FP |
| - | - | - | - | มากับลูก | 28-35 | Findings | 224134001 | FP | FP | 0.0 | FP | FP |
| TC | 37-38 | Laboratory | 121868005 | TC | 37-38 | Procedure | 412808005 | FN&FP | FN&FP | 0.0 | FN&FP | TP (412808005 is child concept of 121868005) |
| LDL | 48-50 | Laboratory | 113079009 | ค่าLDL | 45-50 | Laboratory | 113079009 | FN&FP | TP | 0.5 (3/6) | FN&FP | TP |
| HbA1C | 56-60 | Laboratory | 43396009 | HbA | 56-58 | Procedure | 43396009 | FN&FP | FN&FP | 0.6 (3/5) | FN&FP | TP |
| FBS | 66-68 | Laboratory | 271062006 | FBS | 66-68 | Laboratory | 52302001 | TP | TP | 0.0 | FN&FP | TP (52302001 is parent concept of 271062006) |

Example sentence: “DLP on simvas 10 1*1 วันนี้มากับลูก TC 223, ค่าLDL 135 HbA1C 5.8 FBS 96”, TP: true positive, FP: false positive, FN: false negative
