# Supplementary material for: A framework for structuring Thai medical documents using systematized nomenclature of medicine clinical terms and large language models
Source: PLOS Digit Health. 2026 Oct 30;5(10):e0001791. doi: 10.1371/journal.pdig.0001791 (PMC13722323; doi:10.1371/journal.pdig.0001791)
Supplement: Supplementary file 11 [file pdig.0001791.s011.docx]

S11 Table. Prompt for LLM selection

| Structure | Actual prompt |
| --- | --- |
| Context | You are a clinical language model. Your function is to map a medical entity to the most precise SNOMED CT concept. |
| Task & Input | Given an extracted medical entity, the context it appeared in the record, and 5 SNOMED CT candidate snippets, choose the one snippet that best matches the entity.  Entity: **"{entity}"**  Context: **{context}**  Options:  1. **{doc1}**  2. **{doc2}**  3. **{doc3}**  4. **{doc4}**  5. **{doc5}**  Respond with only the number (1–5). No extra text. |
