# Supplementary material for: A framework for structuring Thai medical documents using systematized nomenclature of medicine clinical terms and large language models
Source: PLOS Digit Health. 2026 Oct 30;5(10):e0001791. doi: 10.1371/journal.pdig.0001791 (PMC13722323; doi:10.1371/journal.pdig.0001791)
Supplement: Supplementary file 12 [file pdig.0001791.s012.docx]

S12 Table. Pseudo-code of Intersection over Union (IoU) calculation

| Line | Pseudo-code |
| --- | --- |
| 1 2 3 4 5 6 7 8 9  10  11 12 13 14 15 16 17 18 19 20 21 | **procedure** COMPUTE_IOU(*G, P*)  total_iou = 0  evaluated_docs = 0  **for** each *doc* in aligned document indices of *G* and *P* **do**  *C_{doc}* = unique concept IDs appearing in *G[doc]* or *P[doc]*  **if** *C_{doc}* is empty then continue  concept_sum = 0  **for** each *cid* in *C_{doc}* **do**  *G_{doc,cid}* = character offsets covered by spans with concept *cid* in *G[doc]*  *P_{doc,cid}* = character offsets covered by spans with concept *cid* in *P[doc]*  intersection = \|*G_{doc,cid}* ∩ *P_{doc,cid}*\|  union = \|*G_{doc,cid}* ∪ *P_{doc,cid}*\|  **if** *union > 0* then  concept_sum = concept_sum + Intersection / union  **end if**  **end for**  total_iou = total_iou + concept_sum / \|*C_{doc}*\|  evaluated_docs = evaluated_docs + 1  **end for**  **return** total_iou / evaluated_docs **end procedure** |

cid: concept identification number, G: Gold entity, P: Predicted entity
