# Supplementary material for: A framework for structuring Thai medical documents using systematized nomenclature of medicine clinical terms and large language models
Source: PLOS Digit Health. 2026 Oct 30;5(10):e0001791. doi: 10.1371/journal.pdig.0001791 (PMC13722323; doi:10.1371/journal.pdig.0001791)
Supplement: Supplementary file 13 [file pdig.0001791.s013.docx]

Annotation Guidelines

# Section I: Annotation Task Overview

Annotation Tool

The tools being used for this annotation task are

1. Doccano: an open-source web-based application for text annotation.
2. Snap2SNOMED: an online platform offered by SNOMED International.

There are **TWO** steps of annotation:

1. Entity annotation: label mention of entities (words or phrases) in each medical document, to the best of your knowledge, and assign entity type for each entity
2. Linking to SNOMED CT: Map the entities with the appropriate SNOMED CT concept

Annotation Steps

**STEP 1**: Entity span annotation

1. Once installed, start Doccano. You will be asked for login information.
2. Once you login, click on the project available to you. This will load all the documents available to you.
3. Click “annotation” on the left panel. Then, start annotate.
4. To annotate, click on the first character of entity and move to the last character of entity span.
5. Select the entity type from the dropdown list.
   a. The instructions on how to use the tool can be found here.
   b. If you are uncertain about the entity span and entity type annotation, please refer to **examples in the following section.**

**STEP 2**: Entity Linking to SNOMED CT

6. Go to Snap2SNOMED platform. You will be asked for login information to Confluence account. The login account can be obtained from SNOMED International.
7. Once you login, click on the project available to you. This will load all the documents available to you.
8. On the right upper card, select your role. Then, start mapping.
9. To map each source term (local term) to target (SNOMED CT concept), click on the PLUS sign on the last column of each row.
   a. The instruction manual on how to use the tool can be found here.
   b. If you are unsure about mapping. Please refer to **examples in the following section.**

-------------------------------------------------------------------------------------------------------------------------------

# Section II: Span annotation

1) Entity spans and entity types: Is the detected text (the colors are different for each entity type) considered to be the concept. If this is correct, go to the next step in the flow chart, if it is not correct - go immediately to the next annotation in that document (automatic), or submit this document if there is nothing else to check inside of it.
2) There are **FOUR** main entity types: findings, laboratory, medication, and procedure. Try to annotate entities with the four main types first. Only when it is not possible to annotate with these types, there are **FOUR** more subtypes: score, anatomy, observable, and event. In total, **EIGHT** entity types.

3) Example of span annotation and entity type assignment.

| Text with span annotated | Entity type |
| --- | --- |
| อาการสบายดี ไม่เหนื่อย | findings |
| HbA1C 5.39 | Laboratory  **No lab value should be annotated |
| Mx start atrovastatin 10 mg | Medication  **annotate only the medication name |
| adviseคุมอาหาร F/U 6 mo | procedure |
| Thai Cv risk >10% | score |
| เริ่มปวดจากนิ้วโป้งเท้าซ้าย | findings & anatomy |
| if BP >130/80 ให้ amlodipine (5) 1 tab oral | observable& medication |
| เคยนั่ง MC กระเทือนแล้วปวด | Event & findings |

4) To use Doccano, login using provided username and password.

5) Select the project named “SNOMED DATA 220”, then on the left side bar select “Dataset”
6) Start annotation by click and slide on the entity you want to annotate and select entity type on the dropdown list.

Common entity annotation problems you might find and the solutions

- Nested entities

Try to keep the semantic first.

Findings in many anatomical concepts: “abdominal and chest pain” annotate abdominal (anatomy), chest (anatomy), and pain (finding)

- Unclear type

Try searching for concept in SNOMED CT first.

Procedure vs Laboratory: See the contextual information. If there are laboratory results reported, label as Laboratory and map to “measurement of …” in SNOMED CT. If the entity is procedural in nature, label as procedure.

- One word, two or more SNOMED concept

Frontotemporal lobes -> annotate into separated SNOMED CT concept

Fronto = Frontal lobe and temporal lobes = Temporal lobe

# Section III: Entity Linking to SNOMED CT

7) Entity linking: this is done through Snap2SNOMED. After the annotation using Doccano is done, data processing is needed to transform json file into tabular data and upload into Snap2SNOMED. Then, each entity will be mapped by one author and one reviewer, as in the flow diagram.

8) Map strength is needed to be defined. The definition of each strength is provided below, as well as some examples.

| Map strength | Description |
| --- | --- |
| Equivalent | The definitions of the concepts mean the same thing (including when structural implications of meaning are considered)  (i.e. extensionally identical). |
| Broader | The target mapping is broader in meaning than the source concept. |
| Narrower | The target mapping is narrower in meaning than the source concept. |
| Inexact | The target mapping overlaps with the source concept, but both source and target cover additional meaning, or the  definitions are imprecise and it is uncertain whether they have the same boundaries to their meaning. |
| No match | There is no match for this concept in the target code system. |

| Term | Map strength | Target (SNOMED CT) |
| --- | --- | --- |
| valvular heart disease | Equivalent | Heart valve disorder (disorder) |
| ckd stage 4 | Equivalent | Chronic kidney disease stage 4 (disorder) |
| ตาพร่า | Equivalent | Blurring of visual image (finding) |
| multiple gallstone | Broader | Gallbladder calculus (disorder) |
| น้ำมูกใส | Broader | Nasal discharge (finding) |
| STEMI | Narrower | Acute ST segment elevation myocardial infarction (disorder) |
| ร้าวลงขา | Narrower | Pain radiating to left leg (finding) |
| full rom | Inexact | Range of joint movement (observable entity) |
| เข้าตา | Inexact | Feeling of sand or foreign body in eye (finding) |

9) In case the map strength is not “Equivalent”, the discussion with research team will be initiated to properly deal with these instances. Re-annotation and re-mapping may be needed.

Help

If any questions arise or if any problems are encountered during the use of the program, please contact your primary investigator.

nat.tag@mahidol.ac.th
